# Rare mutation-dominant compound *EGFR*-positive NSCLC is associated with enriched kinase domain-resided variants of uncertain significance and poor clinical outcomes

**DOI:** 10.1186/s12916-023-02768-z

**Published:** 2023-02-24

**Authors:** Weixin Zhao, Ailing Song, Yang Xu, Qian Wu, Cuicui Liu, Jiani C. Yin, Qiuxiang Ou, Xue Wu, Yang Shao, Xinmin Zhao

**Affiliations:** 1 Department of Radiation Oncology, Fudan University Shanghai Cancer Center, Shanghai, 200032 China; 2 Department of Oncology, Shanghai Medical College, Fudan University, Shanghai, 200032 China; 3 Institute of Thoracic Oncology, Fudan University, Shanghai, 200032 China; 4 Department of Respiratory Medicine, Wuxi Branch of Ruijin Hospital, Shanghai Jiao Tong University, Wuxi, Jiangsu, 214145 China; 5 Geneseeq Research Institute, Nanjing Geneseeq Technology Inc, Nanjing, 210032 Jiangsu China; 6 School of Public Health, Nanjing Medical University, Nanjing, 210029 Jiangsu China; 7 Department of Thoracic Medical Oncology, Fudan University Shanghai Cancer Center, No. 255 Dong’An Road, Shanghai, 200032 China

**Keywords:** Compound *EGFR* mutations, Non-small cell lung cancer, Tyrosine kinase inhibitors, Precision medicine, Resistant mechanism

## Abstract

**Background:**

Compound epidermal growth factor receptor (*EGFR*) mutations are less responsive to tyrosine kinase inhibitors (TKIs) than single *EGFR* mutations in non-small cell lung cancer (NSCLC). However, the detailed clinical characteristics and prognosis of various compound *EGFR* mutations remain to be elucidated.

**Methods:**

We retrospectively studied the next-generation sequencing (NGS) data of treatment-naïve tumors from 1025 NSCLC patients with compound *EGFR* mutations, which were sub-categorized into different combinations of common mutations (19-Del and *EGFR* exon 21 p.L858R), rare mutations, and variants of uncertain significance (VUSs). Prognosis and drug resistance to first-line TKIs were analyzed in 174 and 95 patients, respectively.

**Results:**

Compound *EGFR* mutations were enriched with *EGFR* exon 21 p.L858R and rare mutations, but not 19-Del (*P* < 0.001). The common + rare and rare + rare subtypes had fewer concurrent mutations in the PI3K pathway (*P* = 0.032), while the rare + rare and common + VUSs subtypes showed increased association with smoking- and temozolomide-related mutational signatures, respectively (*P* < 0.001). The rare mutation-dominant subtypes (rare + VUSs and rare + rare) had the worst clinical outcomes to first-line TKIs (*P* < 0.001), which was further confirmed using an external cohort (*P* = 0.0066). VUSs in the rare + VUSs subtype selectively reside in the EGFR kinase domain (*P* < 0.001), implying these tumors might select additional mutations to disrupt the regulation/function of the kinase domain.

**Conclusions:**

Different subtypes of compound *EGFR* mutations displayed distinct clinical features and genetic architectures, and rare mutation-dominant compound *EGFR* mutations were associated with enriched kinase domain-resided VUSs and poor clinical outcomes. Our findings help better understand the oncogenesis of compound *EGFR* mutations and forecast prognostic outcomes of personalized treatments.

**Supplementary Information:**

The online version contains supplementary material available at 10.1186/s12916-023-02768-z.

## Background

Lung cancer is the second most frequent cancer and the leading cause of cancer-related death worldwide [1]. Non-small cell lung cancer (NSCLC) is the major type of lung cancer, and around 14–38% of NSCLC patients harbor genetic alterations in epidermal growth factor receptor (*EGFR*) [2], with the incidence of *EGFR* mutations higher in East Asian patients than in Caucasian patients [3, 4]. Short in-frame deletions in exon 19 (19-Del) and point mutations in *EGFR* exon 21 p.L858R are the most common activating mutations in *EGFR*, accounting for approximately 90% of all *EGFR* mutations in NSCLC [5, 6]. EGFR tyrosine kinase inhibitors (TKIs) have shown profound clinical benefits and are thus used as the first-line treatment in *EGFR*-mutated NSCLC patients [7–12]. Besides 19-Del and *EGFR* exon 21 p.L858R, extensive research has uncovered a wide array of rare *EGFR* activating or resistant mutations in NSCLC, including *EGFR* exon 18 p.G719X, *EGFR* exon 20 p.S768I, *EGFR* exon 21 p.L861Q, *EGFR* exon 20 p.T790M, and *EGFR* exon 20 insertions (20ins). Qin et al. found that *EGFR* 20ins had at least 80 different insertion patterns, and lung cancer patients with *EGFR* 20ins showed different clinical responses to various EGFR TKIs [13]. The *EGFR* exon 20 p.T790M mutation confers drug resistance to first-generation EGFR TKIs, and it has been shown to occur in 1–2% of treatment-naïve *EGFR*-mutated NSCLC patients [14, 15]. In addition to these well-studied common and rare *EGFR* mutations, *EGFR* variants of uncertain significance (VUS) were observed in lung cancer patients, but the clinical relevance and TKI sensitivity of these VUSs are largely unknown [16, 17].

Although the majority of *EGFR*-positive NSCLC patients harbor a single *EGFR* mutation, recent advances in next-generation sequencing (NGS) technologies have revealed that around 10% of patients harbor compound *EGFR* mutations, defined by the presence of double or multiple distinct *EGFR* genetic alterations at baseline [18–20]. Several groups reported that patients with compound *EGFR* mutations tended to be less responsive to TKI therapies than those with a single *EGFR* mutation [21–24]. Furthermore, researchers found that the different types of *EGFR* compound mutations might be associated with distinct treatment efficacies [18, 19]. Despite the potential clinical implications of *EGFR* compound mutations, most of the previous studies were based on limited patient cohorts, so it is imperative to perform large-scale analyses to gain a deeper insight into the complexity and diversity of compound *EGFR* mutations in NSCLC. In the present study, we retrospectively studied the NGS data of treatment-naïve tumor samples from 8485 *EGFR*-mutated NSCLC patients, of whom 1025 had compound *EGFR* mutations. We explored the clinical characteristics and genetic architecture of different types of compound *EGFR* mutations, as well as their responses to EGFR TKIs and the associated drug-resistant mechanisms.

## Methods

### Patients and sample collection

Qualified NGS data from a total of 1025 NSCLC patients harboring compound *EGFR* mutations at baseline from Fudan University Shanghai Cancer Center and Wuxi Branch of Ruijin Hospital were collected as part of the routine diagnosis and treatment. This study was approved by the Ethics Committee of the Fudan University Shanghai Cancer Center, Shanghai Cancer Center Institutional Review Board (SCCIRB), and in accordance with the Declaration of Helsinki (ethics approval number: 2004216–19-2005). Targeted NGS tests were performed in a CLIA-certified and CAP-accredited clinical testing laboratory (Nanjing Geneseeq Technology Inc., Nanjing, China) from April 2016 to October 2020. Of these, 305 were sequenced using a target panel covering 14 key lung cancer-related genes (TETRADECAN™, Geneseeq Technology Inc.) [25], 312 were sequenced by a 139 lung cancer gene panel (PULMOCAN™, Geneseeq Technology Inc.) [26], and 408 were profiled by pan-cancer gene panel covering 425 cancer-relevant genes (GENESEEQPRIME™, Geneseeq Technology Inc.) [27]. Specifically, 5 to 10 mL of peripheral blood was collected from each patient in EDTA-coated tubes (BD Biosciences). Plasma was extracted within 2 h of blood collection and shipped to the central testing laboratory within 48 h. Tumor purity of formalin-fixed paraffin-embedded (FFPE) tumor tissue blocks/sections or fresh tumor tissues was confirmed by the pathologists from the centralized clinical testing center. Written consent was collected from each patient.

### DNA extraction, quantification, and library preparation

DNA extraction, quantification, and library preparation were performed as previously described [28]. In brief, FFPE samples were de-paraffinized with xylene, and DNA was extracted using the QIAamp DNA FFPE Tissue Kit (Qiagen) according to the manufacturer’s protocols. Genomic DNA from fresh tumor tissue was extracted using the DNeasy Blood & Tissue Kit (Qiagen) according to the manufacturer’s protocols. Peripheral blood samples were centrifuged at 1800* g* for 10 min. Then, the plasma was isolated for extraction of cfDNA and the genomic DNA of white blood cells in sediments served as normal controls. The circulating nucleic acid kit (Qiagen, Germany) was used to purify cfDNA from the plasma. The genomic DNA from white blood cells was extracted using the DNeasy Blood and Tissue Kit (Qiagen). Genomic DNA was qualified using a Nanodrop2000 (Thermo Fisher Scientific, Waltham, MA), and cfDNA fragment distribution was analyzed on a Bioanalyzer 2100 using the High Sensitivity DNA Kit (Agilent Technologies, Santa Clara, CA). All DNA was quantified using the dsDNA HS assay kit on a Qubit 3.0 fluorometer (Life Technology, USA) according to the manufacturer’s recommendations. Sequencing libraries were prepared using the KAPA Hyper Prep kit (KAPA Biosystems) with an optimized manufacturer’s protocol and sequenced as previously described [28].

### Data processing

The mean coverage depth was 1402 × for tissue samples, 5655 × for cfDNA samples, and 162 × for matched control samples. Sequencing data were processed as previously described [28]. In brief, mutation calling Trimmomatic was used for FASTQ file quality control, and leading/trailing low-quality (quality reading below 20) or N bases were removed. Qualified reads were mapped to the reference human genome hg19 using Burrows-Wheller Aligner with default parameters, and Genome Analysis Toolkit (GATK 3.4.0) was employed to apply the local realignment around indels and base quality score recalibration. Picard was used to remove PCR duplicates, and samples with mean dedup depth < 30 × were removed. VarScan2 was employed for the detection of single-nucleotide variations (SNVs) and insertion/deletion mutations. SNVs were filtered out if the mutant allele frequency (MAF) was less than 1% for tumor tissue and 0.3% for plasma samples. Variants were further filtered with the following parameters: (i) minimum read depth = 20, (ii) minimum base quality = 15, (iii) minimum variant supporting reads = 5, (iv) variant supporting reads mapped to both strands, (v) strand bias no greater than 10%, (vi) if present in > 1% population in the 1000 Genomes Project or the Exome Aggregation Consortium (ExAC) 65,000 exomes database, and (vii) filtered by an internally collected list of recurrent sequencing errors using a normal pool of 100 samples. Parallel sequencing of matched white blood cells from each patient was performed to further remove sequencing artifacts, germline variants, and clonal hematopoiesis. The copy number alterations were analyzed as previously described [29, 30]. The tumor purities were first estimated using ABSOLUTE [31]. Somatic copy number alteration events were assigned based on sample-ploidy values calculated in the FACETS algorithm [32]. Loss-of-heterozygosity (LOH) was also calculated using FACETS and determined using the minor copy number estimates of each segment for genes in the targeted panel. The minor copy number is by definition 0 in a LOH event [33, 34]. Structural variants were detected using FACTERA with default parameters [35]. The fusion reads were further manually reviewed and confirmed on Integrative Genomics Viewer (IGV).

Tumor mutational burden (TMB, mutation per Megabase) was determined based on the number of somatic base substitutions and indels in the targeted regions of the gene panel covering 0.85 Mb of coding genome, excluding known driver mutations as they are over-represented in the panel. Chromosome instability score (CIS) was defined as the proportion of the genome with aberrant (purity-adjusted segment-level copy number ≥ 3 or ≤ 1) segmented copy number [36].

### Mutation signature analysis

The samples with the number of synonymous/non-synonymous mutations of ≥ 5 were included for mutation signature analysis [37], which was conducted using the “maftools” and “sigminer” R packages. Based on the description of the 30 mutational signatures listed on the COSMIC website (https://cancer.sanger.ac.uk/signatures/signaturesv2/), we classified the signatures into 10 groups, including age (COSMIC1), APOBEC (COSMIC2 and COSMIC13), BRCA (COSMIC3), smoking (COSMIC4), dMMR (COSMIC6, COSMIC15, COSMIC20, and COSMIC26), ultraviolet (COSMIC7), immunoglobulin (COSMIC9), POLE (COSMIC10), temozolomide (COSMIC11), and others (the rest of the signatures). The contribution of each signature was the proportion of the selected signature over all the detected signatures in that specific patient, which was calculated based on previous literature [38–40].

### Statistical analysis

Kaplan–Meier survival curve was used to analyze the progression-free survival (PFS) of various patient groups, and the statistical difference was analyzed using the log‐rank test. Fisher’s exact test was used to test the categorical variables. The Kruskal–Wallis test was conducted to compare multiple groups. Statistical analyses were performed using the R (v4.1.0), and a two-sided *P*-value of < 0.05 was considered to be statistically significant (*P < 0.05, **P < 0.01, ***P < 0.001).

## Results

### Patient characteristics and study plan

**Fig. 1:**
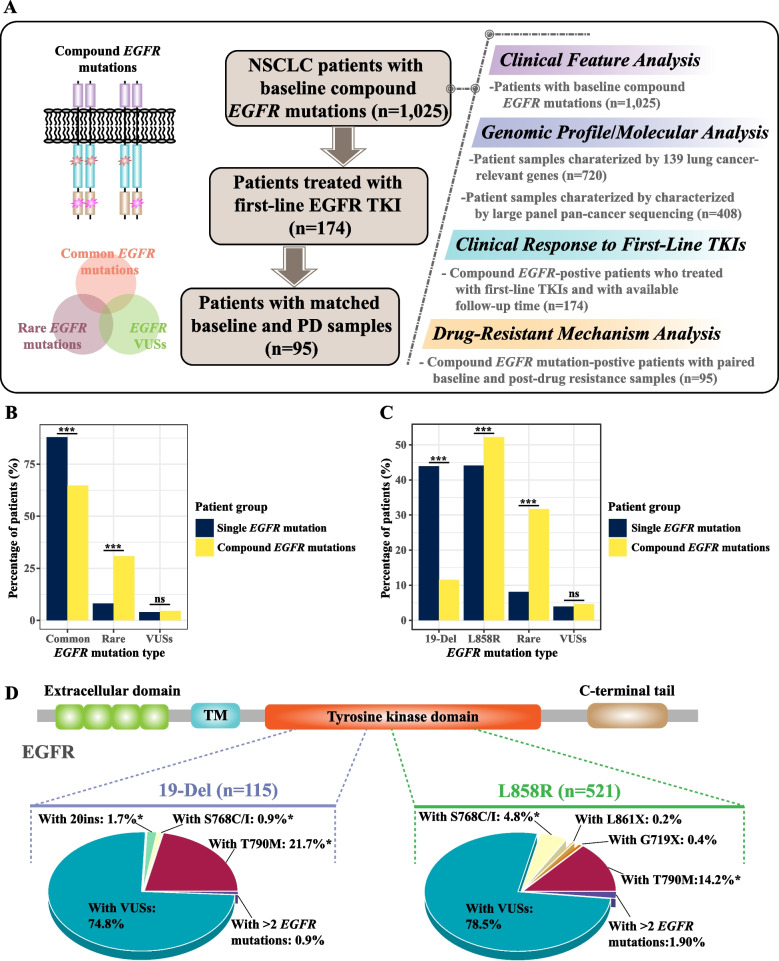
Compound *EGFR* mutation-positive patients had fewer *EGFR* 19-Del mutations and more L858R and rare *EGFR* mutations. **A** The flowchart of the study. **B** Comparing the percentage of patients with single *EGFR* mutation (*n* = 7460) and compound *EGFR* mutations (*n* = 1025) according to their *EGFR* mutation type. Based on the dominant *EGFR* mutations, patients with compound *EGFR* mutations were divided into common (i.e., common + common, common + rare, or common + VUSs), rare (i.e., rare + rare or rare + VUSs), and VUSs (i.e., VUSs + VUSs) groups. **C** Comparing the percentage of patients with single *EGFR* mutation (*n* = 7460) and compound *EGFR* mutations (*N* = 998) according to their *EGFR* mutation type. Patients with concurrent L858R and 19-Del (*n* = 27) were not included in the analysis. **D** The difference of the accompanied *EGFR* mutations between 19-Del and L858R-containing compound *EGFR* mutations. Patients with concurrent L858R and 19-Del (*n* = 27) were not included in the analysis. NGS, next-generation sequencing; VUS, variants of uncertain significance; TM, transmembrane domain

A total of 1025 (12.1%, 1025/8485) patients harbored compound *EGFR* mutations at baseline, that is, two or more distinct *EGFR* mutations were concomitantly detected in a single tumor sample. We sub-categorized compound *EGFR* mutations into different combinations of common *EGFR* mutations (i.e., *EGFR* 19-Del and *EGFR* exon 21 p.L858R), rare *EGFR* mutations (i.e., *EGFR* exon 18 p.G719X, *EGFR* exon 20 p.S768I, *EGFR* exon 21 p.L861Q, *EGFR* exon 20 p.T790M, and *EGFR* 20ins), and/or VUSs. Of the 1025 patients, 570 (55.6%) were older than 60 years, and more than half of the patients (57.8%) were females (Additional file 1: Table S1). The majority of the patients (83.1%) were diagnosed with lung adenocarcinoma (ADC), while other patients had lung squamous cell carcinoma (SCC), adenosquamous carcinoma of the lung (ASC), or unknown histologic subtypes (Additional file 1: Table S1). Clinical features, such as programmed death-ligand 1 (PD-L1) expression, disease stage, and tumor mutation burden (TMB) were also available for 20–40% of the patients (Additional file 1: Table S1). Based on the NGS and clinical data from the 1025 patients, we aimed to investigate compound *EGFR* mutations from various aspects, including the correlation between different types of compound *EGFR* mutations and the clinical/molecular features, as well as delineating therapeutic response to different first-line EGFR TKIs and potential resistant mechanisms, using patients with available post-TKI follow-up information (*n* = 174) and patients with paired baseline and progressive disease (PD) samples (*n* = 95), respectively (Fig. 1A).

### Distinct association between compound *EGFR* mutation subtype and basic clinical features

Among the 1025 compound *EGFR* mutation-positive patients, only 27 (2.6%) harbored multiple (> 2) *EGFR* mutations while 97.4% of the patients had dual *EGFR* mutations (Table 1 and Additional file 1: Fig. S1A). As shown in Additional file 1: Table S2, the presence of multiple *EGFR* mutations was significantly associated with higher TMB (*P* = 0.034). For patients with double *EGFR* mutations, the most frequent combination was common *EGFR* mutation plus VUSs (common + VUSs; 48.2%), followed by rare *EGFR* mutation plus VUSs (rare + VUSs; 17.2%), common + rare (12.7%), and rare + rare (12.6%) (Table 1). In contrast, the common + common (i.e., 19-Del + p.L858R) combination was extremely rare, accounting for only 2.3% of the patients (Table 1).

**Table 1:** The *EGFR* mutation types among the 1025 lung cancer patients with baseline compound *EGFR* mutations

Characteristics	Number of patients, *n* (%)
**Dual *EGFR* mutations**	**998 (97.4%)**
Common + common	24 (2.3%)
Common + rare	130 (12.7%)
Common + VUSs	495 (48.2%)
Rare + VUSs	176 (17.2%)
Rare + rare	129 (12.6%)
VUSs + VUSs	44 (4.3%)
**> 2 *EGFR* mutations**	**27 (2.6%)**
Common + common + rare	2 (0.2%)
Common + common + VUSs	1 (0.1%)
Common + rare + VUSs	2 (0.2%)
Common + rare + rare	2 (0.2%)
Common + VUSs + VUSs	7 (0.7%)
Rare + VUSs + VUSs	9 (0.9%)
Rare + rare + VUSs	1 (0.1%)
Common + common + rare + VUSs	1 (0.1%)
Rare + VUSs + VUSs + VUSs	1 (0.1%)
VUSs + VUSs + VUSs + VUSs	1 (0.1%)

Several clinical features, including age, sex, and TMB, were differentially associated with the type of dual *EGFR* mutations (Additional file 1: Table S3). Specifically, the rare + VUSs subtype was more likely to occur in younger patients (≤ 60 years old) whereas the co-occurrence of *EGFR* 19-Del and *EGFR* exon 21 p.L858R mutations was more frequent in older patients (> 60 years old); in addition, the common + VUSs subtype was more often observed in male patients, and the VUSs + VUSs subtype happened more in patients with higher mutational loads (Additional file 1: Table S3). We also compared compound *EGFR* mutation-positive patients based on whether or not harboring a common *EGFR* mutation. Around two-thirds of these patients (64.7%) were positive for common *EGFR* mutations, and they were more likely to be female and PD-L1 negative (Additional file 1: Table S4). Overall, the different subtypes of compound *EGFR* mutations demonstrated distinct preferences for certain clinical features in NSCLC patients.

### Fewer *EGFR* 19-Del and more *EGFR* exon 21 p.L858R and rare *EGFR* mutations in patients with compound *EGFR* mutations

In order to compare the difference in *EGFR* mutational frequency between patients with single *EGFR* mutation and those with compound *EGFR* mutations, we categorized compound mutation-positive patients according to the priority from common mutations to rare mutations to VUSs. Therefore, based on the highest priority *EGFR* mutation, patients with compound *EGFR* mutations can be divided into three groups, including common (i.e., common + common, common + rare, or common + VUSs), rare (i.e., rare + rare or rare + VUSs), and VUSs (i.e., VUSs + VUSs). Intriguingly, compared with patients with single *EGFR* mutations, compound *EGFR* mutation-positive patients had fewer common and more rare *EGFR* mutations (Fig. 1B). In compound mutation-positive patients with only one common mutation, we performed comparisons between those with *EGFR* 19-Del and *EGFR* exon 21 p.L858R. The lowered incidence of common mutations in compound *EGFR* (64.7% vs 88.0%, *P* < 0.0001) was mainly due to a decrease in the frequency of *EGFR* 19-Del (11.5% vs 43.9%, *P* < 0.0001), whereas *EGFR* exon 21 p.L858R was more common compared with patients with single *EGFR* mutations (52.2% vs 44.1%, *P* < 0.0001; Fig. 1C). In addition, *EGFR* 19-Del and *EGFR* exon 21 p.L858R also differed in their concomitant *EGFR* mutations. *EGFR* 19-Del was more frequently accompanied by baseline mutations such as *EGFR* exon 21 p.T790M and *EGFR* 20ins (*P* = 0.045 and 0.0029, respectively), while *EGFR* exon 21 p.L858R more often co-existed with *EGFR* exon 20 p.S768C/I (*P* = 0.056) (Fig. 1D).

**Fig. 2:**
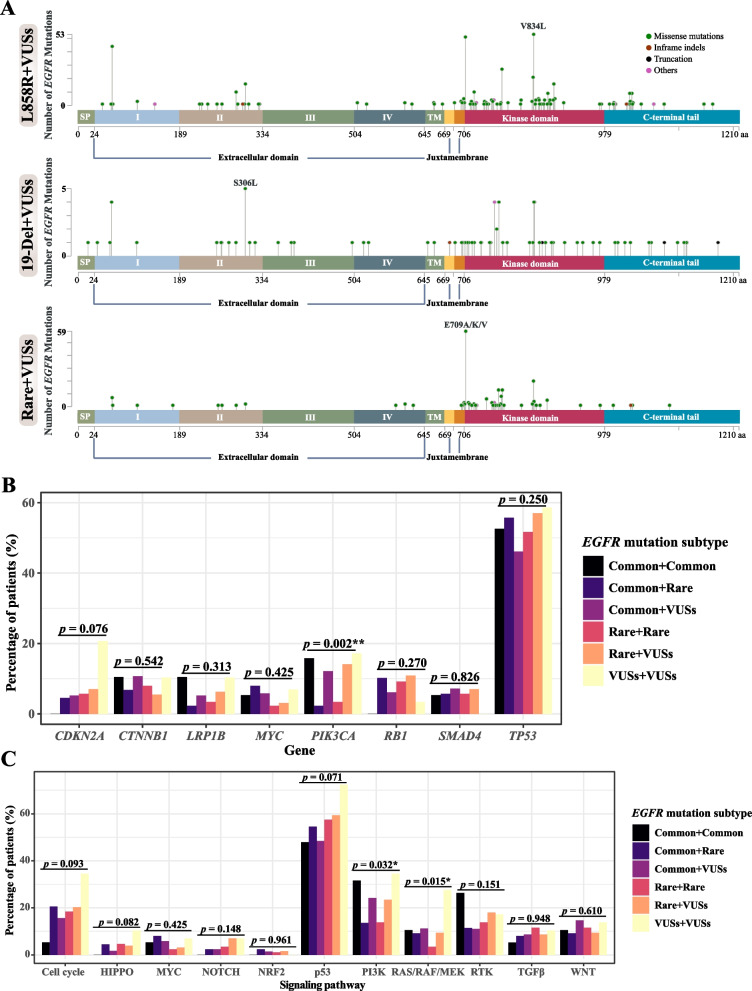
The molecular and genetic characteristics of different types of compound *EGFR* mutations. **A** The lollipop plots of *EGFR* VUSs from various VUS-containing compound *EGFR* mutations, including L858R + VUSs (*n* = 416), 19-Del + VUSs (*n* = 86), and rare + VUSs (*n* = 185). **B** The percentage of patients with various mutated genes stratified by different compound *EGFR* mutation types. Patients’ samples that were characterized by targeted NGS of 139 key lung cancer-related genes were included in the analysis (*n* = 720). **C** The percentage of patients with various altered signaling pathways stratified by different compound *EGFR* mutation types. Patients’ samples that were characterized by targeted NGS of 139 key lung cancer-related genes were included in the analysis (*n* = 720). The Kruskal–Wallis test was conducted to compare multiple groups. *P*-value of < 0.05 was considered to be statistically significant (*P < 0.05, **P < 0.01, ***P < 0.001). SP, signal peptide; TM, transmembrane domain

*EGFR* VUSs were the commonest co-occurring mutations for both *EGFR* exon 21 p.L858R and *EGFR* 19-Del (Fig. 1D). As the function of most VUSs was largely unknown, we evaluated VUSs based on their locations in different EGFR protein domains, including the extracellular domain, transmembrane domain (TM), juxtamembrane domain (JM), kinase domain (KD), and C-terminal tail (Fig. 2A). Intriguingly, the rare + VUSs subtype was highly enriched for KD-located VUSs than other VUS-containing compound *EGFR* mutation subtypes (Fisher’s exact test *P* < 0.001; Fig. 2A and Additional file 1: Table S5), implying the potential importance of additional KD aberrations to reinforce the oncogenic activities of rare *EGFR* mutations.

### Genomic characteristics of different types of compound *EGFR* mutations

A total of 720 patients had baseline tumor samples genetically profiled for 139 key lung cancer-related genes, including *EGFR* (see the “Methods” section for more details), which enabled the investigation of concurrent genetic alterations. *TP53* (50.1%) was the most frequently mutated gene, followed by *PIK3CA* (10.6%), *CTNNB1* (8.9%), and *RB1* (7.9%), across the 720 patients (Additional file 1: Fig. S1B). The frequencies of *PIK3CA* mutations among different compound *EGFR* mutation subtypes were not uniformly distributed (Fig. 2B). Particularly, patients with common + rare and rare + rare subtypes had lower frequencies of *PIK3CA* mutations (Fig. 2B). Similarly, the PI3K pathway was under-represented in the common + rare and rare + rare groups (Fig. 2C and Additional file 1: Table S6). In addition, the rare + rare group also had fewer mutations in genes in the RAS/RAF/MEK pathway (Fig. 2C). In contrast, patients with the VUSs + VUSs subtype tended to have the highest proportion of aberrations in almost all the tested oncogenic pathways (Fig. 2C).

**Fig. 3:**
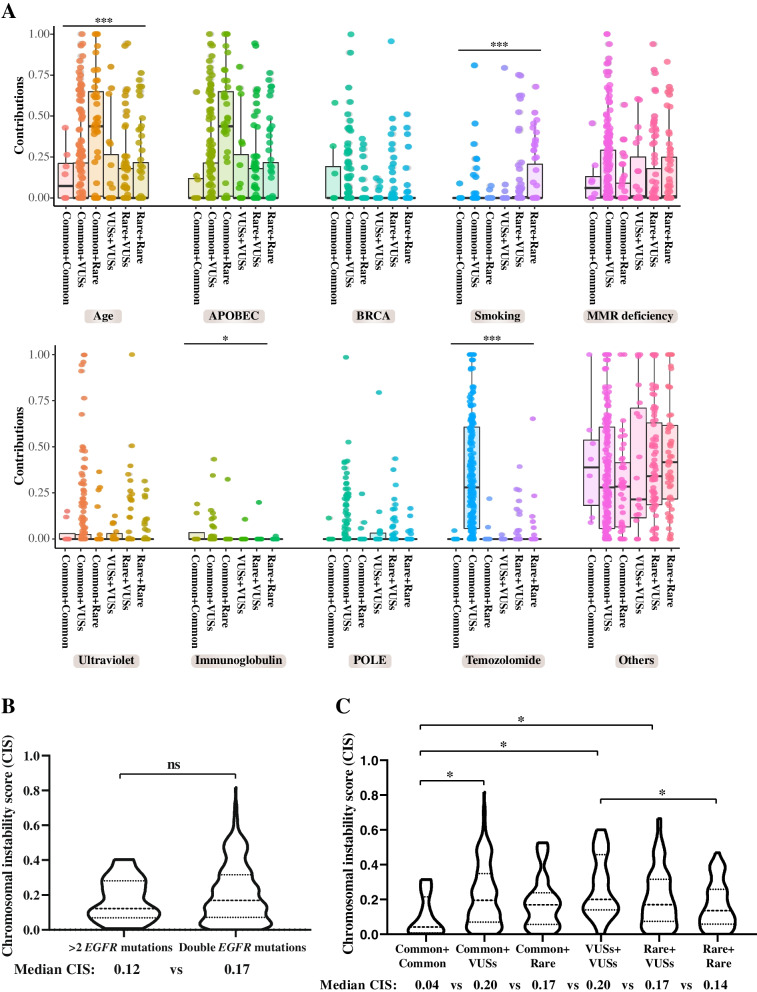
Mutational signature and chromosomal instability of different types of compound *EGFR* mutations. **A** The mutational signature analysis for patients with different types of compound *EGFR* mutations. Patients whose baseline tumor tissue samples were characterized by large panel targeted sequencing of 425 cancer-relevant genes were included in the analysis (*n* = 408). The contribution of each signature was the proportion of the selected signature over all the detected signatures in that specific patient. The Kruskal–Wallis test was conducted to compare multiple groups. The chromosomal instability score in patients with double vs multiple *EGFR* mutations (**B**) or in patients with different types of compound *EGFR* mutations (**C**). Patients whose baseline tumor tissue samples were characterized by large panel targeted sequencing of 425 cancer-relevant genes were included in the analysis (*n* = 408). *P*-value of < 0.05 was considered to be statistically significant (*P < 0.05, **P < 0.01, ***P < 0.001). CIS, chromosomal instability score

Of the 720 patients, 408 underwent large panel targeted sequencing of 425 cancer-relevant genes, including the abovementioned 139 lung cancer-related genes and 286 genes that are frequently mutated in cancers. We performed mutational signature and chromosome instability analyses based on previous studies [41, 42]. An increased number of compound *EGFR* mutations showed little association with the mutational signature (Additional file 1: Fig. S2). On the other hand, the type of compound *EGFR* mutations demonstrated a significant relationship with mutational signatures of age, smoking, immunoglobulin, and temozolomide (Fig. 3A). Particularly, the common + rare subtype displayed more age-related signature, the common + VUSs and rare + rare subtypes were more likely to be associated with the smoking signature, and the common + VUSs subtype also had higher immunoglobulin- and temozolomide-related signatures (Fig. 3A). In terms of chromosome instability, patients with double and multiple *EGFR* mutations had comparable chromosomal instability scores (CISs) (Fig. 3B), whereas patients with the common + common subtype tended to have lower CIS than those with other compound *EGFR* mutation subtypes (Fig. 3C).

### Prognosis of compound *EGFR* mutation-positive patients in response to first-line *EGFR* TKIs

**Fig. 4:**
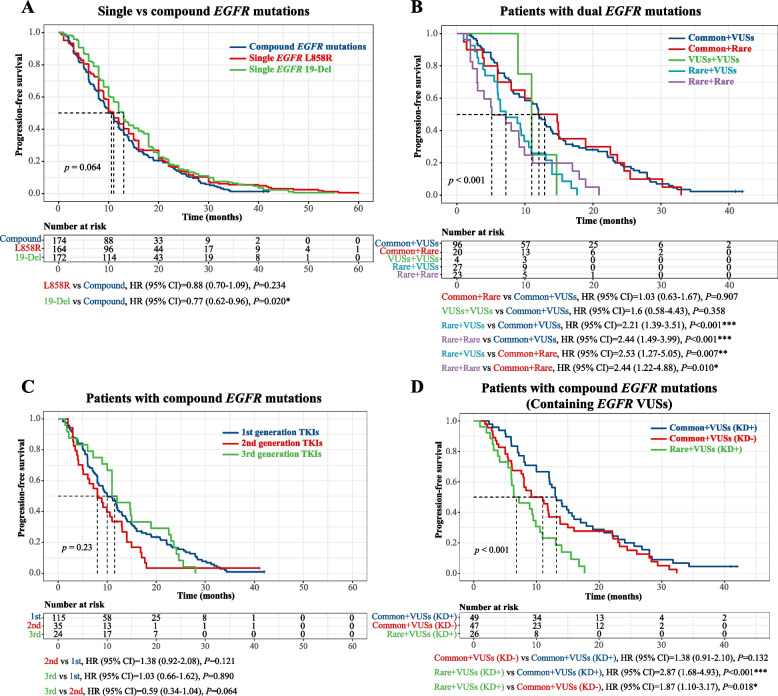
The correlation between the type of compound *EGFR* mutations and patients’ prognosis to first-line EGFR TKIs. **A** Kaplan–Meier curve of progression-free survival in NSCLC patients in strata of the number of *EGFR* mutations. **B** Kaplan–Meier curve of progression-free survival in dual *EGFR* mutation-positive patients in the strata of the various combination of *EGFR* mutations. One patient with the common + common subtype was not included in the analysis. **C** Kaplan–Meier curve of progression-free survival in compound *EGFR* mutation-positive patients in the strata of various generations of EGFR TKIs. **D** Kaplan–Meier curve of progression-free survival in compound *EGFR* mutation-positive patients who harbored *EGFR* VUSs, and these patients were in the strata of different types of compound *EGFR* mutations, as well as the location of the VUSs, which can be inside the EGFR kinase domain (KD +) or outside the EGFR kinase domain (KD −). One patient with the rare + VUSs (KD −) subtype was not included in the analysis. Log‐rank test with *P*-value < 0.05 was considered to be statistically significant (*P < 0.05, **P < 0.01, ***P < 0.001)

Next, we investigated the first-line TKI response in 174 compound *EGFR* mutation-positive patients who had available follow-up data. Consistent with previous research, compound *EGFR* mutations were associated with worse progression-free survival (PFS) than single *EGFR* mutations, and to a higher extent when compared with single *EGFR* 19-Del mutation (Fig. 4A). As only three out of 174 patients had more than 2 *EGFR* mutations, we mainly focused our analysis on those with double *EGFR* mutations. As shown in Fig. 4B, the type of dual *EGFR* mutations had a significant impact on PFS, with common *EGFR* mutation-containing subtypes (common + X) associating with improved PFS than the rare *EGFR* mutation-dominant (rare + VUSs and rare + rare) subtypes (*P* < 0.001). The poor clinical outcome of rare *EGFR* mutation-dominant subtypes was further validated using an external cohort of 22 compound *EGFR* mutation-positive NSCLC patients obtained from the Memorial Sloan Kettering Cancer Center (MSKCC) database (Additional file 1: Fig. S3A). We also divided all patients by the type of first-line EGFR TKIs they received, and the second-generation TKI treatment showed a trend toward having the worst PFS (*P* = 0.23; Fig. 4C).

Subgroup survival analyses comparing different types of dual *EGFR* mutations were performed. Patients were subdivided into common *EGFR* mutation-containing subtypes (common + X), rare *EGFR* mutation-dominant subtypes (rare + VUSs and rare + rare), and VUS-containing subtypes (any subtypes that contain VUSs). Among patients with common *EGFR* mutation-containing subtypes, neither the type of common *EGFR* mutations nor the kind of first-line TKIs had any significant effects on PFS (Additional file 1: Fig. S3B-D). Notably, patients with the 19-Del + X and the L858R + X subtype showed differential survival outcomes to first-line second-generation TKIs, with the 19-Del + X group having a better response and L858R + X displaying unfavorable outcomes. However, the results did not reach statistical significance due to the limited sample size of the subgroups (Additional file 1: Fig. S3E,F). For patients with rare *EGFR* mutation-dominant subtypes, their PFS could not be further stratified by either mutation subtypes or the specific TKI treatments (Additional file 1: Fig. S4A,B). Lastly, we studied patients with VUS-containing subtypes based on the sites of VUSs on EGFR protein, that is, within KD (KD +) versus outside KD (KD −), and we found that the location of VUSs itself could not effectively separate responders from non-responders (Additional file 1: Fig. S5A). However, upon co-analysis with the other *EGFR* mutation, patients with the rare + VUSs (KD +) subtype had significantly shorter PFS than those with the common + VUSs (KD +) subtype (*P* < 0.001) or those with the common + VUSs (KD −) subtype (*P* < 0.001) (Fig. 4D). Only one patient had the rare + VUSs (KD −) subtype and was not included in the Kaplan–Meier analysis in Fig. 4D; nevertheless, this patient had a PFS of 14 months, which was also significantly better than the median PFS (mPFS) of 6.8 months for patients with the rare + VUSs (KD +) subtype. In addition, we studied the impact of specific types of TKIs in patients with VUS-containing subtypes. The third-generation TKIs tended to be associated with better and worse PFS in VUS (KD +) patients and VUS (KD −) patients, respectively, although neither result reached statistical significance due to the limited patient number (Additional file 1: Fig. S5B,C).

### Resistant mechanisms in patients with paired baseline and PD samples

**Fig. 5:**
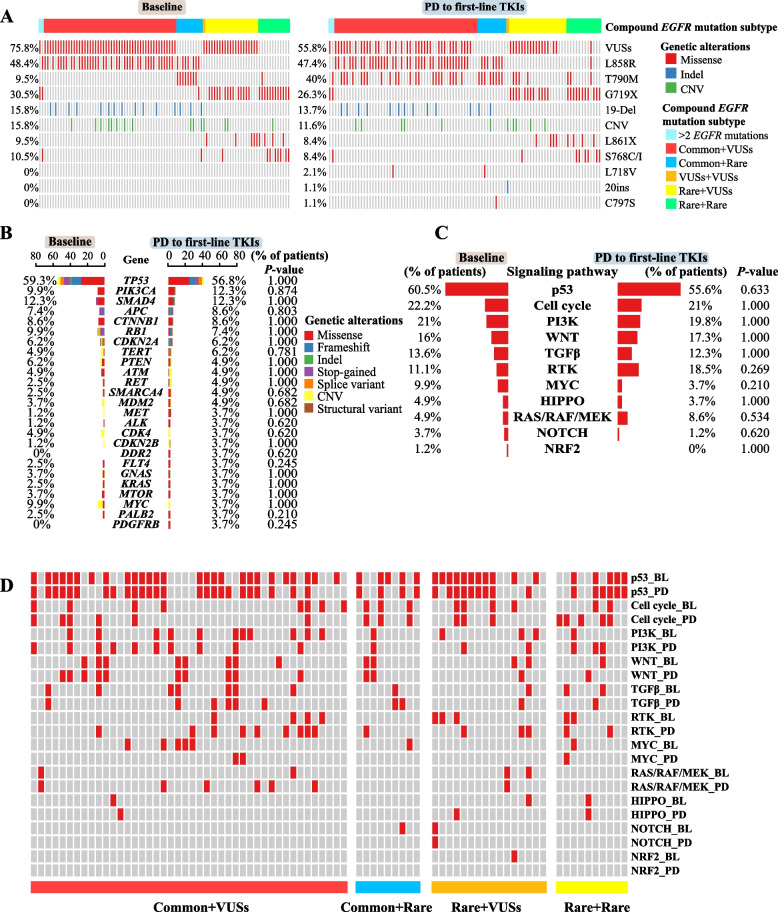
Drug-resistant mechanism analysis using patients with paired baseline and PD samples (*n* = 95). **A** The comparison of *EGFR* mutation status between paired baseline and PD samples. Each column represented a sample derived from a patient, and the two oncoprint plots (i.e., baseline vs PD to first-line TKIs) used the same order to arrange the paired patient samples. The frequency of mutated genes (**B**) or altered signaling pathways (**C**) between the baseline samples and PD samples. **D** The status of aberrant signaling pathways between the baseline and the paired PD samples, stratified by different compound *EGFR* mutation subtypes. BL, baseline; PD, progressive disease

In order to understand the EGFR TKI-resistant mechanisms, we studied 95 compound *EGFR* mutation-positive patients who had paired baseline and PD NGS data. *EGFR* exon 20 p.T790M was the most prevalent *EGFR*-resistant mutation to first-line TKIs, ranging from 9.5% in the baseline samples to 40% in the PD samples (Fig. 5A), and the majority of the acquired *EGFR* exon 20 p.T790M mutation (22/29; 75.9%) occurred in patients with the common + VUSs subtype (*P* < 0.001, Additional file 1: Table S7). Additional gained *EGFR* mutations in PD samples were also observed, including *EGFR* exon 18 p.L718V, *EGFR* 20ins, and *EGFR* exon 20 p.C797S (Fig. 5A). We further investigated the potential off-target resistance mechanisms (Additional file 1: Fig. S6) and found no genetic alterations or signaling pathways that were significantly different between the baseline and the PD samples (Fig. 5B, C). Notably, when comparing patients based on the baseline compound *EGFR* mutation type, the common + VUSs subtype acquired more mutations in the RAS/RAF/MEK pathway than other subtypes (11.4% vs 0%, *P* = 0.266, Fig. 5D). Overall, the different compound *EGFR* mutation types might rely on differential TKI-resistant mechanisms, with the common + VUSs subtype specifically enriched for *EGFR* exon 20 p.T790M and/or other RAS/RAF/MEK pathway-related mutations.

## Discussion

We performed a large-scale retrospective study of 1025 NSCLC patients who harbored baseline compound *EGFR* mutations. Intriguingly, compound *EGFR* mutations had a significantly higher frequency of *EGFR* exon 21 p.L858R and rare *EGFR* mutations and a dramatically lower rate of *EGFR* 19-Del mutation than single *EGFR* mutation. Different types of compound *EGFR* mutations demonstrated distinct subtypes of mutated genes, aberrant signaling pathways, mutational signatures, and chromosomal instability. Notably, the rare *EGFR* mutation-dominant subtypes were associated with significantly shorter FPS. In addition, VUSs in the rare + VUSs subtype were more likely to locate at the EGFR kinase domain, and patients with rare + VUSs (KD +) had worse PFS than those with other VUS-containing subtypes. In terms of TKI-resistant mechanism, the common + VUSs subtype was highly enriched for *EGFR* exon 20 p.T790M and/or other RAS/RAF/MEK pathway-related mutations. Therefore, different compound *EGFR* mutation subtypes had distinct clinical/genetic characteristics and therapeutic responses.

The first-generation EGFR TKIs (e.g., gefitinib and erlotinib) are ATP‑competitive small molecules that reversibly target the EGFR tyrosine kinase domain. Despite its significant clinical benefits when compared with chemotherapies in NSCLC patients, drug resistance inevitably developed [43]. To overcome the resistance to first-generation TKIs, the second-generation TKIs (e.g., afatinib and dacomitinib), which are irreversible inhibitors, were designed. Although second-generation TKIs generally showed improved EGFR inhibition, they also exhibited high potency against wild-type EGFR, leading to lower maximum dose tolerance, more adverse events, and limited clinical utilities [44, 45]. One of the most common resistance mechanisms against both the first- and second-generation TKIs is *EGFR* exon 20 p.T790M mutation [46–48]. The gatekeeper hypothesis suggests that the steric hindrance between the methionine residue on the gatekeeper side chain of *EGFR* exon 20 p.T790M and the aniline moiety of first-generation TKIs is the underlying mechanism of the drug resistance, although other putative mechanisms have been proposed, including elevated ATP-binding affinity for *EGFR* exon 20 p.T790M, changes in the catalytic domain, and variations in the conformational dynamics [49, 50]. In our study, we found that a significant proportion of patients with common + VUSs subtype (44%) acquired *EGFR* exon 20 p.T790M mutation after EGFR TKI treatments, but not for other *EGFR* subtypes. Because the percentage of acquiring *EGFR* exon 20 p.T790M is similar between the common + VUSs subtype in our study and other studies using patients with a single *EGFR* common mutation [51], we speculate that the common + VUSs subtype might resemble the function of a single *EGFR* common mutation. In particular, the *EGFR* VUSs in the common + VUSs subtype might be passenger mutations and did not contribute to the oncogenic activation of EGFR. In contrast, some *EGFR* compound mutation subtypes (e.g., rare + rare and rare + VUSs) are less likely to acquire *EGFR* exon 20 p.T790M, implying that these subtypes might rewire the signaling network to make them prone to utilize other resistance mechanisms to bypass first- and second-generation TKIs. The third-generation TKIs, especially osimertinib, demonstrated satisfactory efficacy against *EGFR* exon 20 p.T790M. Osimertinib formed irreversible covalent bonds with the cysteine 797 residue in the ATP-binding site, and it exhibited selective potency against the mutant EGFR rather than wild-type EGFR, resulting in its accelerated approval by US Food and Drug Administration to treat *EGFR*-mutated NSCLC [52]. One patient in our cohort gained *EGFR* exon 20 p.C797S mutation after first-line TKIs and became resistant to osimertinib. This patient might be treated with TKI combinations or next-generation TKIs to overcome this resistance mutation [53].

Around 12.1% of *EGFR*-positive NSCLC patients in our cohort harbored compound *EGFR* mutations, which is consistent with previous studies [18–20]. Only around 2% of all compound *EGFR* mutation-positive patients had more than 2 baseline *EGFR* mutations, and these patients generally had high tumor mutation loads. Kauffmann-Guerrero et al. reported that compound *EGFR* mutations were more often observed in patients with a smoking history [22]. Although our patient cohort did not have complete records of the patient’s smoking status, the mutational signature results suggested that not all subtypes of compound *EGFR* mutations had the same level of smoking-related signatures, with common + VUSs and rare + rare subtypes being more likely to occur in smokers than other subtypes. Additionally, Kim et al. found that compound *EGFR* mutations were frequently co-mutated with some actionable genes, such as *ALK* rearrangement, *KRAS* mutation, and *PIK3CA* mutations [23]. We also detected multiple co-mutated genes, which exhibited distinct subtypes according to the specific type of compound *EGFR* mutations. Particularly, unlike other compound *EGFR* mutations, the rare + rare subtype had a significantly low frequency of mutations in the PI3K and RAS/RAF/MEK signaling pathways, implying that tumors harboring double rare *EGFR* mutations might less rely on these oncogenic pathways. On the other hand, the VUSs + VUSs subtype had the highest mutational frequency in almost all the tested oncogenic pathways. This indicates that many of the detected *EGFR* VUSs might have little or very mild oncogenic activities, and tumors harboring the VUSs + VUSs subtype had to heavily depend on other oncogenic mutations for tumorigenesis and maintenance.

Another interesting observation of our study is that compound *EGFR* mutations had a much lower frequency of *EGFR* 19-Del and a significantly higher frequency of *EGFR* exon 21 p.L858R than the single *EGFR* mutation. The two types of common *EGFR* mutations also had different preferences in the co-existed *EGFR* mutations. Furthermore, the *EGFR* 19-Del + X subtype and *EGFR* exon 21 p.L858R + X subtype had opposite trends in the therapeutic response to second-generation TKIs. Multiple previous studies on single *EGFR* mutation have found that *EGFR* 19-Del and *EGFR* exon 21 p.L858R demonstrated different clinical features and treatment outcomes. Hong’s group reported that patients with a single *EGFR* 19‑Del mutation had significantly improved clinical outcomes than patients with a single *EGFR* exon 21 p.L858R mutation following first‑line TKI, but not first‑line chemotherapy or second‑line TKI [54]. NSCLC patients with *EGFR* 19-Del also had a higher risk of lymph node metastasis than those with *EGFR* exon 21 p.L858R [55]. Despite the clinical difference between *EGFR* exon 21 p.L858R and *EGFR* 19-Del, the underlying mechanism is still elusive. Sordella et al. discovered that *EGFR* exon 21 p.L858R and *EGFR* 19-Del had differential levels of EGFR autophosphorylation on some specific sites, which may affect their drug sensitivity to TKIs [56]. Nevertheless, future studies are needed to elucidate the distinguishing preference of *EGFR* exon 21 p.L858R and *EGFR* 19-Del in compound *EGFR* mutations.

We found that patients with compound *EGFR* mutations tended to be less responsive to EGFR TKIs than those with single *EGFR* mutation, especially the patients with single *EGFR* 19-Del, which is consistent with previous studies [21–24]. Additionally, we discovered that different subtypes of compound *EGFR* mutations were also significantly associated with patient’s prognosis to first-line TKIs. Specifically, the presence of a common mutation in compound *EGFR* mutations can sufficiently predict prognosis, regardless of the type and location of the other *EGFR* mutation. However, for rare *EGFR* mutation-containing patients, their prognosis is likely to highly rely on the type of mutation combinations. In particular, rare + common was associated with good PFS, rare + VUSs (KD −) might be related to good to intermediate PFS, while rare + rare and rare + VUSs (KD +) are likely to associate with short PFS. Therefore, both the type of *EGFR* mutations (common vs rare vs VUSs) and the specific combination of compound mutations might contribute to the overall prognosis of NSCLC patients.

The common + common subtype was extremely rare, accounting for only 2.3% of patients in our cohort. Given that common *EGFR* mutations could efficiently activate EGFR kinase activity and promote tumorigenesis, it is highly unlikely that a single tumor would acquire two *EGFR* common mutations simultaneously. As a result, we suspect that the two different *EGFR* common mutations might mainly reside in different tumor cells. In other words, we think those patients might have two subclones of cancer cells, one is driven by *EGFR* exon 21 p.L858R and the other is driven by *EGFR* 19-Del, and both of them are likely to be sensitive to EGFR TKIs. For the common + rare and common + VUSs subtypes, the two *EGFR* mutations could be either in the same or in different tumor cells. However, if some common and rare *EGFR* mutations are in the same cancer cells, they might interfere with the response to certain EGFR TKIs. For example, Yu et al. found that if lung cancer patients had co-occurred baseline common *EGFR* mutation and baseline *EGFR* exon 20 p.T790M, they had poor responses to first-generation TKIs [57]. Indeed, several previous studies reported that common *EGFR* mutations and *EGFR* exon 20 p.T790M were almost always in cis configurations in order to confer resistance to first-generation EGFR TKIs [58]. Additionally, we found that rare *EGFR* mutations were specifically enriched for *EGFR* VUS (KD +) mutations. We speculate that *EGFR* VUSs (KD +) and rare *EGFR* mutations are within the same cancer cell or even on the same allele, and the additional KD aberrations from the VUSs might help reinforce the oncogenic activities of rare *EGFR* mutations. Strikingly, we found that patients with the rare + VUSs (KD +) subtype are generally associated with a poorer prognosis than those with other subtypes, which further implies that they might reside in the same cancer cells to drive tumorigenesis and/or tumor progression. Nevertheless, our NGS results were not ideal to elucidate whether the compound *EGFR* mutations were from the same cancer cell/DNA allele or not. Among the 1025 patients in our cohort, the compound *EGFR* mutations of 282 patients were on the same exon. We then analyzed whether the mutations were on the same sequencing read (i.e., the same allele) or not. Strikingly, in 98.9% (279/282) of cases, the compound *EGFR* mutations were located on the same allele, which also infers that they were in the same cancer cell (Additional file 1: Table S8). Future studies using more appropriate approaches (e.g., NGS on multi-site sampling tissues, single-cell sequencing, sequencing complementary DNAs, long-read sequencing, or fluorescent in situ hybridization) are necessary to further check the cis/trans configuration and cellular distribution of compound mutations.

There were several limitations of our study. Firstly, a large proportion of patients had missing clinical information, including the PD-L1 expression and disease stages, which can potentially impede thorough analyses of the correlation between the clinical characteristics and compound *EGFR* mutation subtypes. Secondly, because the tumor samples were collected by different hospitals spanning the past 4.5 years, the samples were generically profiled by 3 different targeted sequencing panels. Fortunately, all 3 targeted sequencing panels were designed and performed by the same sequencing institute. Specifically, all the assay validations were performed using a method-based validation approach to detect a specific type of mutation at a specific sequencing depth under the entire NGS system, and all three sequencing panels showed a similar capacity to detect mutations (cross-panel accuracy > 97%). Therefore, the result of overlapping genes from the three sequencing panels is comparable. Lastly, only 95 patients who had paired baseline and PD samples were available for drug resistance analyses, and future studies with larger patient sizes are necessary to fully elucidate the differential resistant mechanisms for various compound *EGFR* mutations.

## Conclusions

In conclusion, by performing a large-scale analysis in 1025 compound *EGFR* mutation-positive NSCLC patients, we found that different subtypes of compound *EGFR* mutations were associated with distinct demographic features, co-mutated genes, mutational signatures, and chromosomal instability levels, as well as distinguishing prognosis to first-line EGFR TKIs. Our study helps better understand the clinical characteristics of compound *EGFR* mutations and emphasizes the importance of determining the specific types of *EGFR* mutations, which can potentially direct prognosis prediction and provide personalized treatments to NSCLC patients.

## Supplementary Information

**Additional file 1:** **Table S1.** The demographic and clinical characteristics ofthe 1,025 lung cancer patients with baseline compound *EGFR* mutations. **Table S2.** The correlation of clinical features with thenumber concurrent *EGFR* mutations. **Table S3.** The correlation of clinicalfeatures with the subtype of compound *EGFR*mutations. **Table S4.** The correlationof clinical features with the presence or absence of common *EGFR* mutations. **Table S5.** The enrichment of different subtypes of compound *EGFR* mutations in various domains ofEGFR protein. **Table S6.** The involvedgenes of each path during the pathway analysis. EGFR has been excluded from RTKpathway analysis. **Table S7.** The gainof *EGFR* exon 20 p.T790M mutation inprogressive disease (PD) samples after front-line EGFR TKI treatment inpatients with different subtypes of compound *EGFR* mutations. **Table S8.**The distribution of compound *EGFR*mutations for 282 patients with their compound *EGFR* mutations on the same exon. **Fig. S1.** The type of compound *EGFR*mutations and the concurrent genetic alterations. **Fig. S2.** The mutational signature analysis for patients withdifferent numbers of *EGFR* mutations. **Fig. S3.** The correlation between thecommon *EGFR* mutation-containingsubtype and patients’ prognosis to first-line EGFR TKIs. **Fig. S4.** The correlation between the rare *EGFR* mutation-dominant subtype and patients’ prognosis tofirst-line EGFR TKIs. **Fig. S5.** Thecorrelation between the *EGFR*VUSs-containing subtype and patients’ prognosis to first-line EGFR TKIs. **Fig. S6.** The difference of the geneticprofile between the baseline sample and the paired PD samples.

## Data Availability

The data generated in this study are available within the article and its supplementary data files. The raw sequencing data are not publicly available due to privacy or ethical restrictions but are available upon reasonable request from the corresponding author.

## Abbreviations

19-Del: Short in-frame deletions in exon 19
20ins: Exon 20 insertions
ADC: Lung adenocarcinoma
ASC: Adenosquamous carcinoma of the lung
CIS: Chromosome instability score
*EGFR*: Epidermal growth factor receptor
ExAC: Exome aggregation consortium
FFPE: Formalin-fixed paraffin-embedded
IGV: Integrative genomics viewer
JM: Juxtamembrane domain
KD: Kinase domain
LOH: Loss-of-heterozygosity
MAF: Mutant allele frequency
mPFS: Median progression-free survival
NGS: Next-generation sequencing
NSCLC: Non-small cell lung cancer
PD: Progressive disease
PD-L1: Programmed death-ligand 1
PFS: Progression-free survival
SCC: Lung squamous cell carcinoma
SNVs: Single-nucleotide variations
TKI: Tyrosine kinase inhibitor
TM: Transmembrane domain
TMB: Tumor mutational burden
VUS: Variant of uncertain significance
