# Supplementary material for: Rare mutation-dominant compound EGFR-positive NSCLC is associated with enriched kinase domain-resided variants of uncertain significance and poor clinical outcomes
Source: BMC Med. 2023 Feb 24;21:73. doi: 10.1186/s12916-023-02768-z (PMC9960474; doi:10.1186/s12916-023-02768-z)
Supplement: Supplementary file 1 — Additional file 1: Table S1. The demographic and clinical characteristics ofthe 1,025 lung cancer patients with baseline compound EGFR mutations. Table S2. The correlation of clinical features with thenumber concurrent EGFR mutations. Table S3. The correlation of clinicalfeatures with the subtype of compound EGFRmutations. Table S4. The correlationof clinical features with the presence or absence of common EGFR mutations. Table S5. The enrichment of different subtypes of compound EGFR mutations in various domains ofEGFR protein. Table S6. The involvedgenes of each path during the pathway analysis. EGFR has been excluded from RTKpathway analysis. Table S7. The gainof EGFR exon 20 p.T790M mutation inprogressive disease (PD) samples after front-line EGFR TKI treatment inpatients with different subtypes of compound EGFR mutations. Table S8.The distribution of compound EGFRmutations for 282 patients with their compound EGFR mutations on the same exon. Fig. S1. The type of compound EGFRmutations and the concurrent genetic alterations. Fig. S2. The mutational signature analysis for patients withdifferent numbers of EGFR mutations. Fig. S3. The correlation between thecommon EGFR mutation-containingsubtype and patients’ prognosis to first-line EGFR TKIs. Fig. S4. The correlation between the rare EGFR mutation-dominant subtype and patients’ prognosis tofirst-line EGFR TKIs. Fig. S5. Thecorrelation between the EGFRVUSs-containing subtype and patients’ prognosis to first-line EGFR TKIs. Fig. S6. The difference of the geneticprofile between the baseline sample and the paired PD samples. [file 12916_2023_2768_MOESM1_ESM.docx]

**Table S1** The demographic and clinical characteristics of the 1,025 lung cancer patients with baseline compound *EGFR* mutations.

| **Characteristics** | **Number of patient, n (%)** |
| --- | --- |
| **Age** |  |
| ≤60 | 443 (43.2%) |
| >60 | 570 (55.6%) |
| unknown | 12 (1.2%) |
| **Sex** |  |
| Female | 592 (57.8%) |
| Male | 433 (42.2%) |
| **PD-L1 expression** |  |
| Positive | 76 (7.4%) |
| Negative | 93 (9.1%) |
| Failed | 13 (1.3%) |
| No test | 843 (82.2%) |
| **TMB** |  |
| <10 | 350 (34.1%) |
| ≥10 | 58 (5.7%) |
| Unknown | 617 (60.2%) |
| **Disease stage** |  |
| I | 38 (3.7%) |
| II | 19 (1.9%) |
| III | 32 (3.1%) |
| IV | 175 (17.1%) |
| Unknown | 761 (74.2%) |
| **Pathology** |  |
| ADC | 852 (83.1%) |
| SCC | 17 (1.7%) |
| ASC | 6 (0.6%) |
| Others | 6 (0.6%) |
| Unknown | 144 (14.0%) |

**Table S2** The correlation of clinical features with the number concurrent *EGFR* mutations.

| **Characteristics** | **Dual *EGFR* mutations (n=998)** | **>2 *EGFR* mutations (n=27)** | ***P*-value** |
| --- | --- | --- | --- |
| **Age (years) , n (%)** |  |  | 0.78 |
| ≤60 | 430 (43.1) | 13 (48.1) |  |
| >60 | 556 (55.7) | 14 (51.9) |  |
| Unknown | 12 (1.2) | 0 (0.0) |  |
| **Sex, n (%)** |  |  | 0.694 |
| Female | 575 (57.6) | 17 (63.0) |  |
| Male | 423 (42.4) | 10 (37.0) |  |
| **Histology, n (%)** |  |  | 0.223 |
| LUAD | 833 (83.5) | 19 (70.4) |  |
| LUSC | 16 (1.6) | 1 (3.7) |  |
| ASC | 6 (0.6) | 0 (0.0) |  |
| Others | 6 (0.6) | 0 (0.0) |  |
| Unknown | 137 (13.7) | 7 (25.9) |  |
| **Disease stage, n (%)** |  |  | 0.89 |
| I-III | 86 (8.6) | 3 (11.1) |  |
| IV | 171 (17.2) | 4 (14.8) |  |
| Unknown | 741 (74.2) | 20 (74.1) |  |
| **PD-L1 expression, n (%)** |  |  | 0.851 |
| Negative | 90 (9.0) | 3 (11.1) |  |
| Positive | 74 (7.4) | 2 (7.4) |  |
| Unknown | 834 (83.6) | 22 (81.5) |  |
| **TMB, n (%)** |  |  | 0.034* |
| ≥10 | 53 (5.3) | 5 (18.5) |  |
| <10 | 342 (34.3) | 8 (29.6) |  |
| Unknown | 603 (60.4) | 14 (51.9) |  |

**Table S3** The correlation of clinical features with the subtype of compound *EGFR* mutations.

| **Characteristics** | **Common+ Common (n=24)** | **Common+ Rare (n=130)** | **Common+ VUSs  (n=495)** | **Rare+ VUSs  (n=176)** | **Rare+ Rare  (n=129)** | **VUSs+ VUSs  (n=44)** | ***P*-value** |
| --- | --- | --- | --- | --- | --- | --- | --- |
| **Age (years), n (%)** |  |  |  |  |  |  | 0.002** |
| ≤60 | 6 (25.0) | 52 (40.0) | 217 (43.8) | 104 (59.1) | 65 (50.4) | 22 (50.0) |  |
| >60 | 18 (75.0) | 76 (58.5) | 272 (55.0) | 69 (39.2) | 64 (49.6) | 21 (47.7) |  |
| Unknown | 0 (0.0) | 2 (1.5) | 6 (1.2) | 3 (1.7) | 0 (0.0) | 1 (2.3) |  |
| **Sex, n (%)** |  |  |  |  |  |  | 0.001** |
| Female | 13 (54.2) | 71 (54.6) | 191 (38.6) | 93 (52.8) | 73 (56.6) | 21 (47.7) |  |
| Male | 11 (45.8) | 59 (45.4) | 305 (61.4) | 83 (47.2) | 56 (43.4) | 23 (52.3) |  |
| **Histology, n (%)** |  |  |  |  |  |  | 0.758 |
| LUAD | 20 (83.3) | 111 (85.4) | 414 (83.6) | 147 (83.5) | 109 (84.5) | 32 (72.7) |  |
| LUSC | 0 (0.0) | 1 (0.8) | 9 (1.8) | 2 (1.1) | 2 (1.6) | 2 (4.5) |  |
| ASC | 0 (0.0) | 0 (0.0) | 2 (0.4) | 2 (1.1) | 1 (0.8) | 1 (2.3) |  |
| VUSs | 0 (0.0) | 0 (0.0) | 3 (0.6) | 1 (0.6) | 2 (1.6) | 0 (0.0) |  |
| Unknown | 4 (16.7) | 18 (13.8) | 67 (13.6) | 24 (13.7) | 15 (11.5) | 9 (20.5) |  |
| **Disease stage, n (%)** |  |  |  |  |  |  | 0.989 |
| I-III | 1 (4.2) | 10 (7.7) | 47 (9.5) | 13 (7.4) | 10 (7.8) | 4 (9.1) |  |
| IV | 3 (12.5) | 25 (19.2) | 80 (16.2) | 33 (18.8) | 23 (17.8) | 7 (15.9) |  |
| Unknown | 20 (83.3) | 95 (73.1) | 368 (74.3) | 130 (73.8) | 96 (74.4) | 33 (75.0) |  |
| **PD-L1 expression, n (%)** |  |  |  |  |  |  | 0.227 |
| Negative | 2 (8.3) | 11 (8.5) | 53 (10.7) | 10 (5.7) | 11 (8.5) | 3 (6.8) |  |
| Positive | 2 (8.3) | 9 (6.9) | 27 (5.5) | 21 (11.9) | 10 (7.8) | 5 (11.4) |  |
| Unknown | 20 (83.3) | 110 (84.6) | 415 (83.8) | 145 (82.4) | 108 (83.7) | 36 (81.8) |  |
| **TMB, n (%)** |  |  |  |  |  |  | 0.043* |
| ≥10 | 2 (8.3) | 3 (2.3) | 22 (4.4) | 10 (5.7) | 9 (7.0) | 7 (15.9) |  |
| <10 | 6 (25.0) | 37 (28.5) | 172 (34.8) | 69(39.2) | 46 (35.6) | 12 (27.3) |  |
| Unknown | 16 (66.7) | 90 (69.2) | 301 (60.8) | 97 (55.1) | 74 (57.4) | 25 (56.8) |  |

**Table S4** The correlation of clinical features with the presence or absence of common *EGFR* mutations.

| **Characteristics** | **With common *EGFR* mutations (n=663)** | **Without common *EGFR* mutations (n=362)** | ***P*-value** |
| --- | --- | --- | --- |
| **Age (years), n (%)** |  |  | 0.687 |
| ≤60 | 280 (42.2) | 163 (45.0) |  |
| >60 | 375 (56.6) | 195 (53.9) |  |
| Unknown | 8 (1.2) | 4 (1.1) |  |
| **Sex, n (%)** |  |  | 0.046* |
| Female | 398 (60.0) | 194 (53.6) |  |
| Male | 265 (40.0) | 168 (46.4) |  |
| **Histology, n (%)** |  |  | 0.529 |
| LUAD | 554 (83.5) | 298 (82.3) |  |
| LUSC | 11 (1.7) | 6 (1.7) |  |
| ASC | 2 (0.3) | 4 (1.1) |  |
| Others | 3 (0.5) | 3 (0.8) |  |
| Unknown | 93 (14.0) | 51 (14.1) |  |
| **Disease stage, n (%)** |  |  | 0.745 |
| I-III | 59 (8.9) | 30 (8.3) |  |
| IV | 109 (16.4) | 66 (18.2) |  |
| Unknown | 495 (74.7) | 266 (73.5) |  |
| **PD-L1 expression, n (%)** |  |  | 0.012* |
| Negative | 66 (10.0) | 27 (7.5) |  |
| Positive | 38 (5.7) | 38 (10.5) |  |
| Unknown | 559 (84.3) | 297 (82.0) |  |
| **TMB, n (%)** |  |  | 0.023* |
| ≥10 | 29 (4.4) | 29 (8.0) |  |
| <10 | 220 (33.2) | 130 (35.9) |  |
| Unknown | 414 (62.4) | 203 (56.1) |  |

**Table S5** The enrichment of different subtypes of compound *EGFR* mutations in various domains of EGFR protein.

| **EGFR domain** | **Compound mutation type (with VUSs)** | **Number of patient, n (%)** | ***P*-value** |
| --- | --- | --- | --- |
| Extracellular  domain | p.L858R+vus | 93 (21.9%) | <0.001*** |
|  | 19del+vus | 25 (29.1%) |  |
|  | rare+vus | 20 (10.6%) |  |
| Transmembrane  and  juxtamembrane | L858R+vus | 16 (3.8%) | 0.222 |
|  | 19del+vus | 6 (7.0%) |  |
|  | rare+vus | 5 (2.6%) |  |
| Kinase domain | L858R+vus | 273 (64.2%) | <0.001*** |
|  | 19del+vus | 42 (48.8%) |  |
|  | rare+vus | 160 (84.7%) |  |
| C-terminal tail | L858R+vus | 43 (10.1%) | <0.001*** |
|  | 19del+vus | 13 (15.1%) |  |
|  | rare+vus | 4 (2.1%) |  |

**Table S6** The involved genes of each path during the pathway analysis. EGFR has been excluded from RTK pathway analysis.

| **Pathway** | **Genes included in the pathway** |
| --- | --- |
| RTK | ERBB2, ERBB3, ERBB4, MET, PDGFRA, FGFR1, FGFR2, FGFR3, FGFR4, KIT, IGF1R, RET, ROS1, ALK, FLT3, NTRK1-3, JAK2 |
| RAS/RAF/MEK | CBL, ERRFI1, ABL1, SOS1, NF1, RASA1, PTPN11, KRAS, HRAS, NRAS, RIT1, ARAF, BRAF, RAF1, RAC1, MAPK1, MAP2K1, MAP2K2 |
| WNT | WIF1, SFRP1-5, RNF43, ZNRF3, FZDs, LRP5/6, DKK1-4, GSK3B, APC, CTNNB1, AXIN1/2, AMER1, TCF7, TCF7L1/2, TLE1-4 |
| HIPPO | DCHS1/2, FAT1/2/3/4, TAOK1/2/3, SAV1, STK3/4, LATS1/2, MOB1A/B, NF2, WWC1, YAP1, TAZ, CRB1/2, PTPN14, CSNK1E/D, TEAD2 |
| NRF2 | KEAP1, CUL3, NFE2L2 |
| TGFβ | TGFBR1/2, ACVR2A/1B, SMAD2, SMAD3, SMAD4 |
| MYC | MYC, MYCN, MYCL, MAX, MGA, MXD1/3/4, MXI1, MNT, MLX, MLXIP, MLXIPL |
| NOTCH | NOV, CNTN6, JAG2, ARRDC1, NOTCH1/2/3/4, DNER, PSEN2, FBXW7, CUL1, MAML3, KAT2B, CREBBP, EP300, NCOR1/2, SPEN, KDM5A, HES-X, HEY-X |
| PI3K | PTEN, PIK3R2, INPP4B, PIK3CA/B, PIK3R1/3, AKT1, AKT2, AKT3, PPP2R1A, STK11, TSC1/2, RHEB, RICTOR, MTOR, RPTOR |
| p53 | MDM2/4, CDKN2A, ATM, CHEK2, RPS6KA3, TP53 |
| Cell cycle | CDKN1A, CDKN1B, CDKN2A, CDKN2B/C, CCNE1, RB1, CCND1/2/3, CDK2, CDK4, CDK6, E2F1/3 |

**Table S7** The gain of *EGFR* exon 20 p.T790M mutation in progressive disease (PD) samples after front-line EGFR TKI treatment in patients with different subtypes of compound *EGFR* mutations.

| **Type of compound EGFR mutations** | **Number of patients** | **Patients with baseline T790M, n (%)** | **Patients with PD T790M, n (%)** | ***P-*value** |
| --- | --- | --- | --- | --- |
| >2 *EGFR* mutations | 2 | 0 (0.0%) | 1 (50%) | ＞0.999 |
| Common+VUSs | 50 | 0 (0.0%) | 22 (44%) | <0.001*** |
| Common+Rare | 10 | 8 (80%) | 8 (80%) | ＞0.999 |
| VUSs+VUSs | 1 | 0 (0.0%) | 0 (0.0%) | ＞0.999 |
| Rare+VUSs | 20 | 0 (0.0%) | 4 (20%) | 0.106 |
| Rare+Rare | 12 | 1 (8.3%) | 3 (25%) | 0.59 |
| **Total** | 95 | 9 (9.5%) | 38 (40%) | <0.001*** |

**Table S8** The distribution of compound *EGFR* mutations for 282 patients with their compound *EGFR* mutations on the same exon.

| **Patient ID** | ***EGFR* genetic alterations** | **Exon** | **Same sequencing read** | **Distance between sites (bp)** |
| --- | --- | --- | --- | --- |
| P1 | p.L858R (c.T2573G) | exon 21 | yes | 25 |
|  | p.L833V (c.T2497G) | exon 21 |  |  |
| P2 | p.L858R (c.T2573G) | exon 21 | yes | 20 |
|  | p.L838V (c.C2512G) | exon 21 |  |  |
| P3 | p.L858R (c.T2573G) | exon 21 | yes | 13 |
|  | p.A871G (c.C2612G) | exon 21 |  |  |
| P4 | p.L861Q (c.T2582A) | exon 21 | yes | 28 |
|  | p.L833W (c.T2498G) | exon 21 |  |  |
| P5 | p.G719S (c.G2155A) | exon 18 | yes | 10 |
|  | p.E709A (c.A2126C) | exon 18 |  |  |
| P6 | p.L858R (c.T2573G) | exon 21 | yes | 24 |
|  | p.V834L (c.G2500C) | exon 21 |  |  |
| P7 | p.H835F (c.CA2503_2504TT) | exon 21 | yes | 2 |
|  | p.L833V (c.T2497G) | exon 21 |  |  |
| P8 | p.V774M (c.G2320A) | exon 20 | yes | 1 |
|  | p.H773L (c.A2318T) | exon 20 |  |  |
| P9 | p.V834L (c.G2500T) | exon 21 | yes | 24 |
|  | p.L858R (c.T2573G) | exon 21 |  |  |
| P10 | p.V834L (c.G2500T) | exon 21 | yes | 24 |
|  | p.L858R (c.T2573G) | exon 21 |  |  |
| P11 | p.L858R (c.T2573G) | exon 21 | yes | 3 |
|  | p.L861M (c.C2581A) | exon 21 |  |  |
| P12 | p.L858R (c.T2573G) | exon 21 | yes | 15 |
|  | p.V843I (c.G2527A) | exon 21 |  |  |
| P13 | p.G719S (c.G2155A) | exon 18 | yes | 10 |
|  | p.E709A (c.A2126C) | exon 18 |  |  |
| P14 | p.G719C (c.G2155T) | exon 18 | yes | 10 |
|  | p.E709A (c.A2126C) | exon 18 |  |  |
| P15 | p.G719S (c.G2155A) | exon 18 | yes | 10 |
|  | p.E709K (c.G2125A) | exon 18 |  |  |
| P16 | p.L858M (c.C2572A) | exon 21 | yes | 3 |
|  | p.L861Q (c.T2582A) | exon 21 |  |  |
| P17 | p.L833V (c.T2497G) | exon 21 | yes | 25 |
|  | p.L858R (c.T2573G) | exon 21 |  |  |
| P18 | p.E709V (c.A2126T) | exon 18 | yes | 10 |
|  | p.G719A (c.G2156C) | exon 18 |  |  |
| P19 | p.L858R (c.T2573G) | exon 21 | yes | 24 |
|  | p.V834L (c.G2500T) | exon 21 |  |  |
| P20 | p.L858R (c.T2573G) | exon 21 | yes | 24 |
|  | p.V834L (c.G2500T) | exon 21 |  |  |
| P21 | p.G719C (c.G2155T) | exon 18 | yes | 10 |
|  | p.E709A (c.A2126C) | exon 18 |  |  |
| P22 | p.G719C (c.G2155T) | exon 18 | yes | 10 |
|  | p.E709A (c.A2126C) | exon 18 |  |  |
| P23 | p.L833V (c.T2497G) | exon 21 | yes | 2 |
|  | p.H835L (c.A2504T) | exon 21 |  |  |
| P24 | p.L833V (c.T2497G) | exon 21 | yes | 25 |
|  | p.L858R (c.T2573G) | exon 21 |  |  |
| P25 | p.V834L (c.G2500T) | exon 21 | yes | 24 |
|  | p.L858R (c.T2573G) | exon 21 |  |  |
| P26 | p.V834L (c.G2500C) | exon 21 | yes | 24 |
|  | p.L858R (c.T2573G) | exon 21 |  |  |
| P27 | p.T854A (c.A2560G) | exon 21 | yes | 4 |
|  | p.L858R (c.T2573G) | exon 21 |  |  |
| P28 | p.V834L (c.G2500T) | exon 21 | yes | 24 |
|  | p.L858R (c.T2573G) | exon 21 |  |  |
| P29 | p.E709A (c.A2126C) | exon 18 | yes | 10 |
|  | p.G719S (c.G2155A) | exon 18 |  |  |
| P30 | p.L747P (c.TT2239_2240CC) | exon 19 | yes | 1 |
|  | p.E746D (c.A2238T) | exon 19 |  |  |
| P31 | p.G719C (c.G2155T) | exon 18 | yes | 10 |
|  | p.E709A (c.A2126C) | exon 18 |  |  |
| P32 | p.L703V (c.C2107G) | exon 18 | yes | 16 |
|  | p.G719A (c.G2156C) | exon 18 |  |  |
| P33 | p.K860I (c.A2579T) | exon 21 | yes | 2 |
|  | p.L858R (c.T2573G) | exon 21 |  |  |
| P34 | p.V834L (c.G2500T) | exon 21 | yes | 24 |
|  | p.L858R (c.T2573G) | exon 21 |  |  |
| P35 | p.V769L (c.G2305T) | exon 20 | yes | 1 |
|  | p.S768I (c.G2303T) | exon 20 |  |  |
| P36 | p.G719S (c.G2155A) | exon 18 | yes | 10 |
|  | p.E709A (c.A2126C) | exon 18 |  |  |
| P37 | p.H773L (c.A2318T) | exon 20 | yes | 1 |
|  | p.V774M (c.G2320A) | exon 20 |  |  |
| P38 | p.L833V (c.T2497G) | exon 21 | yes | 2 |
|  | p.H835L (c.A2504T) | exon 21 |  |  |
| P39 | p.E709V (c.A2126T) | exon 18 | yes | 10 |
|  | p.G719A (c.G2156C) | exon 18 |  |  |
| P40 | p.E709K (c.G2125A) | exon 18 | yes | 10 |
|  | p.G719A (c.G2156C) | exon 18 |  |  |
| P41 | p.L833V (c.T2497G) | exon 21 | yes | 2 |
|  | p.H835L (c.A2504T) | exon 21 |  |  |
| P42 | p.L858R (c.T2573G) | exon 21 | yes | 24 |
|  | p.V834L (c.G2500T) | exon 21 |  |  |
| P43 | p.V834L (c.G2500T) | exon 21 | yes | 24 |
|  | p.L858R (c.T2573G) | exon 21 |  |  |
| P44 | p.L858R (c.T2573G) | exon 21 | yes | 24 |
|  | p.V834L (c.G2500T) | exon 21 |  |  |
| P45 | p.V834L (c.G2500T) | exon 21 | yes | 24 |
|  | p.L858R (c.T2573G) | exon 21 |  |  |
| P46 | p.Q701L (c.A2102T) | exon 18 | yes | 18 |
|  | p.G719A (c.G2156C) | exon 18 |  |  |
| P47 | p.G719C (c.G2155T) | exon 18 | yes | 10 |
|  | p.E709V (c.A2126T) | exon 18 |  |  |
| P48 | p.E709K (c.G2125A) | exon 18 | yes | 10 |
|  | p.G719A (c.2156_2157delinsCA) | exon 18 |  |  |
| P49 | p.L833V (c.T2497G) | exon 21 | yes | 25 |
|  | p.L858R (c.T2573G) | exon 21 |  |  |
| P50 | p.V834L (c.G2500T) | exon 21 | yes | 24 |
|  | p.L858R (c.T2573G) | exon 21 |  |  |
| P51 | p.H835L (c.A2504T) | exon 21 | yes | 2 |
|  | p.L833V (c.T2497G) | exon 21 |  |  |
| P52 | p.S768I (c.G2303T) | exon 20 | yes | 1 |
|  | p.V769L (c.G2305T) | exon 20 |  |  |
| P53 | E709A | exon 18 | yes | 10 |
|  | G719S | exon 18 |  |  |
| P54 | p.L833W (c.T2498G) | exon 21 | yes | 28 |
|  | p.L861Q (c.T2582A) | exon 21 |  |  |
| P55 | p.E709A (c.A2126C) | exon 18 | yes | 10 |
|  | p.G719C (c.G2155T) | exon 18 |  |  |
| P56 | p.E709A (c.A2126C) | exon 18 | yes | 10 |
|  | p.G719S (c.G2155A) | exon 18 |  |  |
| P57 | p.L844V (c.C2530G) | exon 21 | yes | 14 |
|  | p.L858R (c.T2573G) | exon 21 |  |  |
| P58 | p.G719C (c.G2155T) | exon 18 | yes | 10 |
|  | p.E709A (c.A2126C) | exon 18 |  |  |
| P59 | p.L833V (c.T2497G) | exon 21 | yes | 2 |
|  | p.H835L (c.A2504T) | exon 21 |  |  |
| P60 | p.L858R (c.T2573G) | exon 21 | yes | 24 |
|  | p.V834L (c.G2500T) | exon 21 |  |  |
| P61 | p.A871E (c.C2612A) | exon 21 | yes | 13 |
|  | p.L858R (c.T2573G) | exon 21 |  |  |
| P62 | p.H835L (c.A2504T) | exon 21 | yes | 2 |
|  | p.L833V (c.T2497G) | exon 21 |  |  |
| P63 | p.E709K (c.G2125A) | exon 18 | yes | 10 |
|  | p.G719A (c.G2156C) | exon 18 |  |  |
| P64 | p.L858R (c.T2573G) | exon 21 | yes | 4 |
|  | p.T854A (c.A2560G) | exon 21 |  |  |
| P65 | c.2500G>T(p.V834L) | exon 21 | yes | 24 |
|  | c.2573T>G(p.L858R) | exon 21 |  |  |
| P66 | c.2500G>T(p.V834L) | exon 21 | yes | 24 |
|  | c.2573T>G(p.L858R) | exon 21 |  |  |
| P67 | c.2500G>T(p.V834L) | exon 21 | yes | 24 |
|  | c.2573T>G(p.L858R) | exon 21 |  |  |
| P68 | c.2500G>T(p.V834L) | exon 21 | yes | 24 |
|  | c.2573_2574delTGinsGT(p.L858R) | exon 21 |  |  |
| P69 | c.2303G>T(p.S768I) | exon 20 | yes | 6 |
|  | c.2320G>A(p.V774M) | exon 20 |  |  |
| P70 | c.2582T>A(p.L861Q) | exon 21 | yes | 28 |
|  | c.2498T>G(p.L833W) | exon 21 |  |  |
| P71 | c.2573T>G(p.L858R) | exon 21 | yes | 24 |
|  | c.2500G>T(p.V834L) | exon 21 |  |  |
| P72 | c.2156G>C(p.G719A) | exon 18 | yes | 10 |
|  | c.2126A>C(p.E709A) | exon 18 |  |  |
| P73 | c.2500G>T(p.V834L) | exon 21 | yes | 24 |
|  | c.2573T>G(p.L858R) | exon 21 |  |  |
| P74 | c.2155G>T(p.G719C) | exon 18 | yes | 10 |
|  | c.2126A>C(p.E709A) | exon 18 |  |  |
| P75 | c.2156G>C(p.G719A) | exon 18 | yes | 10 |
|  | c.2125G>A(p.E709K) | exon 18 |  |  |
| P76 | c.2500G>T(p.V834L) | exon 21 | yes | 24 |
|  | c.2573T>G(p.L858R) | exon 21 |  |  |
| P77 | c.2573T>G(p.L858R) | exon 21 | yes | 6 |
|  | c.2590G>C(p.A864P) | exon 21 |  |  |
| P78 | c.2573T>G(p.L858R) | exon 21 | yes | 13 |
|  | c.2612C>G(p.A871G) | exon 21 |  |  |
| P79 | c.2555A>C(p.K852T) | exon 21 | yes | 6 |
|  | c.2573T>G(p.L858R) | exon 21 |  |  |
| P80 | c.2504A>T(p.H835L) | exon 21 | yes | 2 |
|  | c.2497T>G(p.L833V) | exon 21 |  |  |
| P81 | c.2528T>C(p.V843A) | exon 21 | yes | 15 |
|  | c.2573T>G(p.L858R) | exon 21 |  |  |
| P82 | c.2156G>C(p.G719A) | exon 18 | yes | 10 |
|  | c.2125G>A(p.E709K) | exon 18 |  |  |
| P83 | c.2573T>G(p.L858R) | exon 21 | yes | 13 |
|  | c.2612C>G(p.A871G) | exon 21 |  |  |
| P84 | c.2126A>C(p.E709A) | exon 18 | yes | 10 |
|  | c.2155G>T(p.G719C) | exon 18 |  |  |
| P85 | c.2497T>G(p.L833V) | exon 21 | yes | 25 |
|  | c.2573T>G(p.L858R) | exon 21 |  |  |
| P86 | c.2125G>A(p.E709K) | exon 18 | yes | 10 |
|  | c.2155G>A(p.G719S) | exon 18 |  |  |
| P87 | c.2579A>T(p.K860I) | exon 21 | yes | 2 |
|  | c.2573T>G(p.L858R) | exon 21 |  |  |
| P88 | c.2125G>A(p.E709K) | exon 18 | yes | 10 |
|  | c.2155G>T(p.G719C) | exon 18 |  |  |
| P89 | c.2612C>A(p.A871E) | exon 21 | yes | 13 |
|  | c.2573T>G(p.L858R) | exon 21 |  |  |
| P90 | c.2612C>G(p.A871G) | exon 21 | yes | 13 |
|  | c.2573T>G(p.L858R) | exon 21 |  |  |
| P91 | c.2155G>T(p.G719C) | exon 18 | yes | 10 |
|  | c.2126A>C(p.E709A) | exon 18 |  |  |
| P92 | c.2155G>A(p.G719S) | exon 18 | yes | 10 |
|  | c.2125G>A(p.E709K) | exon 18 |  |  |
| P93 | c.2573T>G(p.L858R) | exon 21 | yes | 24 |
|  | c.2500G>T(p.V834L) | exon 21 |  |  |
| P94 | c.2500G>C(p.V834L) | exon 21 | yes | 24 |
|  | c.2573T>G(p.L858R) | exon 21 |  |  |
| P95 | c.2318A>T(p.H773L) | exon 20 | yes | 1 |
|  | c.2320G>A(p.V774M) | exon 20 |  |  |
| P96 | c.2573_2574delTGinsGT(p.L858R) | exon 21 | yes | 24 |
|  | c.2500G>T(p.V834L) | exon 21 |  |  |
| P97 | c.2573T>G(p.L858R) | exon 21 | yes | 24 |
|  | c.2500G>T(p.V834L) | exon 21 |  |  |
| P98 | c.2500G>C(p.V834L) | exon 21 | yes | 27 |
|  | c.2582T>A(p.L861Q) | exon 21 |  |  |
| P99 | c.2500G>T(p.V834L) | exon 21 | yes | 24 |
|  | c.2573T>G(p.L858R) | exon 21 |  |  |
| P100 | c.2155G>C(p.G719R) | exon 18 | yes | 10 |
|  | c.2126A>C(p.E709A) | exon 18 |  |  |
| P101 | c.2572C>A(p.L858M) | exon 21 | yes | 3 |
|  | c.2582T>G(p.L861R) | exon 21 |  |  |
| P102 | c.2126A>G(p.E709G) | exon 18 | yes | 10 |
|  | c.2155G>A(p.G719S) | exon 18 |  |  |
| P103 | c.2126A>T(p.E709V) | exon 18 | yes | 10 |
|  | c.2156G>C(p.G719A) | exon 18 |  |  |
| P104 | c.2305G>T(p.V769L) | exon 20 | yes | 1 |
|  | c.2303G>T(p.S768I) | exon 20 |  |  |
| P105 | c.2500G>T(p.V834L) | exon 21 | yes | 24 |
|  | c.2573T>G(p.L858R) | exon 21 |  |  |
| P106 | c.2612C>G(p.A871G) | exon 21 | yes | 13 |
|  | c.2573T>G(p.L858R) | exon 21 |  |  |
| P107 | c.2573T>G(p.L858R) | exon 21 | yes | 24 |
|  | c.2500G>T(p.V834L) | exon 21 |  |  |
| P108 | c.2590G>C(p.A864P) | exon 21 | yes | 6 |
|  | c.2573T>G(p.L858R) | exon 21 |  |  |
| P109 | c.2499_2500delGGinsAT(p.V834L) | exon 21 | yes | 24 |
|  | c.2573T>G(p.L858R) | exon 21 |  |  |
| P110 | c.2612C>G(p.A871G) | exon 21 | yes | 13 |
|  | c.2573T>G(p.L858R) | exon 21 |  |  |
| P111 | c.2579A>T(p.K860I) | exon 21 | yes | 2 |
|  | c.2573T>G(p.L858R) | exon 21 |  |  |
| P112 | c.2573T>G(p.L858R) | exon 21 | yes | 24 |
|  | c.2500G>T(p.V834L) | exon 21 |  |  |
| P113 | c.2573T>G(p.L858R) | exon 21 | yes | 24 |
|  | c.2500G>T(p.V834L) | exon 21 |  |  |
| P114 | c.2497T>G(p.L833V) | exon 21 | yes | 25 |
|  | c.2573T>G(p.L858R) | exon 21 |  |  |
| P115 | c.2154_2155delGGinsTT(p.G719C) | exon 18 | yes | 10 |
|  | c.2126A>T(p.E709V) | exon 18 |  |  |
| P116 | c.2320G>A(p.V774M) | exon 20 | yes | 1 |
|  | c.2318A>T(p.H773L) | exon 20 |  |  |
| P117 | c.2125G>A(p.E709K) | exon 18 | yes | 10 |
|  | c.2156G>C(p.G719A) | exon 18 |  |  |
| P118 | c.2504A>T(p.H835L) | exon 21 | yes | 2 |
|  | c.2497T>G(p.L833V) | exon 21 |  |  |
| P119 | c.2156G>C(p.G719A) | exon 18 | yes | 5 |
|  | c.2140A>G(p.K714E) | exon 18 |  |  |
| P120 | c.2573T>G(p.L858R) | exon 21 | yes | 5 |
|  | c.2588G>A(p.G863D) | exon 21 |  |  |
| P121 | c.2126A>C(p.E709A) | exon 18 | yes | 10 |
|  | c.2155G>A(p.G719S) | exon 18 |  |  |
| P122 | c.2573T>G(p.L858R) | exon 21 | yes | 25 |
|  | c.2499G>C(p.L833F) | exon 21 |  |  |
| P123 | c.2573T>G(p.L858R) | exon 21 | yes | 14 |
|  | c.2530C>G(p.L844V) | exon 21 |  |  |
| P124 | c.2527G>A(p.V843I) | exon 21 | yes | 15 |
|  | c.2573T>G(p.L858R) | exon 21 |  |  |
| P125 | c.2573T>G(p.L858R) | exon 21 | yes | 24 |
|  | c.2500G>T(p.V834L) | exon 21 |  |  |
| P126 | c.2504A>T(p.H835L) | exon 21 | yes | 2 |
|  | c.2497T>G(p.L833V) | exon 21 |  |  |
| P127 | c.2305G>T(p.V769L) | exon 20 | yes | 1 |
|  | c.2303G>T(p.S768I) | exon 20 |  |  |
| P128 | c.2155G>A(p.G719S) | exon 18 | yes | 10 |
|  | c.2126A>C(p.E709A) | exon 18 |  |  |
| P129 | c.2573T>G(p.L858R) | exon 21 | yes | 25 |
|  | c.2497T>G(p.L833V) | exon 21 |  |  |
| P130 | c.2125G>A(p.E709K) | exon 18 | yes | 15 |
|  | c.2170G>A(p.G724S) | exon 18 |  |  |
| P131 | c.2126A>C(p.E709A) | exon 18 | yes | 10 |
|  | c.2156G>C(p.G719A) | exon 18 |  |  |
| P132 | c.340G>A(p.E114K) | exon 3 | yes | 9 |
|  | c.315G>T(p.Q105H) | exon 3 |  |  |
| P133 | c.2573T>G(p.L858R) | exon 21 | yes | 12 |
|  | c.2609A>G(p.H870R) | exon 21 |  |  |
| P134 | c.2497T>G(p.L833V) | exon 21 | yes | 25 |
|  | c.2573T>G(p.L858R) | exon 21 |  |  |
| P135 | c.2582T>A(p.L861Q) | exon 21 | yes | 30 |
|  | c.2492G>A(p.R831H) | exon 21 |  |  |
| P136 | c.2155G>A(p.G719S) | exon 18 | yes | 10 |
|  | c.2126A>C(p.E709A) | exon 18 |  |  |
| P137 | c.2497T>G(p.L833V) | exon 21 | yes | 25 |
|  | c.2573T>G(p.L858R) | exon 21 |  |  |
| P138 | c.2155G>A(p.G719S) | exon 18 | yes | 10 |
|  | c.2126A>T(p.E709V) | exon 18 |  |  |
| P139 | c.2573T>G(p.L858R) | exon 21 | yes | 15 |
|  | c.2618G>A(p.G873E) | exon 21 |  |  |
| P140 | c.2579A>T(p.K860I) | exon 21 | yes | 2 |
|  | c.2573T>G(p.L858R) | exon 21 |  |  |
| P141 | c.2126A>C(p.E709A) | exon 18 | yes | 10 |
|  | c.2156G>C(p.G719A) | exon 18 |  |  |
| P142 | c.2582T>G(p.L861R) | exon 21 | yes | 28 |
|  | c.2499G>T(p.L833F) | exon 21 |  |  |
| P143 | c.2156G>C(p.G719A) | exon 18 | yes | 10 |
|  | c.2125G>A(p.E709K) | exon 18 |  |  |
| P144 | c.2320G>A(p.V774M) | exon 20 | yes | 6 |
|  | c.2303G>T(p.S768I) | exon 20 |  |  |
| P145 | c.2500G>T(p.V834L) | exon 21 | yes | 24 |
|  | c.2573T>G(p.L858R) | exon 21 |  |  |
| P146 | c.2155G>A(p.G719S) | exon 18 | yes | 10 |
|  | c.2125G>A(p.E709K) | exon 18 |  |  |
| P147 | c.2530C>G(p.L844V) | exon 21 | yes | 14 |
|  | c.2573T>G(p.L858R) | exon 21 |  |  |
| P148 | c.2126A>C(p.E709A) | exon 18 | yes | 10 |
|  | c.2155G>T(p.G719C) | exon 18 |  |  |
| P149 | c.2512C>G(p.L838V) | exon 21 | yes | 20 |
|  | c.2573T>G(p.L858R) | exon 21 |  |  |
| P150 | c.2504A>T(p.H835L) | exon 21 | yes | 2 |
|  | c.2497T>G(p.L833V) | exon 21 |  |  |
| P151 | c.2126A>C(p.E709A) | exon 18 | yes | 10 |
|  | c.2155G>A(p.G719S) | exon 18 |  |  |
| P152 | p.L858R (c.T2573G) | exon 21 | yes | 15 |
|  | p.G873E (c.G2618A) | exon 21 |  |  |
| P153 | c.2500G>T(p.V834L) | exon 21 | yes | 24 |
|  | c.2573T>G(p.L858R) | exon 21 |  |  |
| P154 | c.2303G>T(p.S768I) | exon 20 | yes | 1 |
|  | c.2305G>T(p.V769L) | exon 20 |  |  |
| P155 | c.2125G>A(p.E709K) | exon 18 | yes | 10 |
|  | c.2156G>C(p.G719A) | exon 18 |  |  |
| P156 | c.2155G>A(p.G719S) | exon 18 | yes | 10 |
|  | c.2126A>G(p.E709G) | exon 18 |  |  |
| P157 | c.2612C>A(p.A871E) | exon 21 | yes | 13 |
|  | c.2573T>G(p.L858R) | exon 21 |  |  |
| P158 | c.2573T>G(p.L858R) | exon 21 | yes | 24 |
|  | c.2500G>T(p.V834L) | exon 21 |  |  |
| P159 | c.2612C>G(p.A871G) | exon 21 | yes | 13 |
|  | c.2573T>G(p.L858R) | exon 21 |  |  |
| P160 | c.2500G>C(p.V834L) | exon 21 | yes | 24 |
|  | c.2573T>G(p.L858R) | exon 21 |  |  |
| P161 | c.2579A>T(p.K860I) | exon 21 | yes | 2 |
|  | c.2573T>G(p.L858R) | exon 21 |  |  |
| P162 | c.2303G>T(p.S768I) | exon 20 | yes | 6 |
|  | c.2320G>A(p.V774M) | exon 20 |  |  |
| P163 | c.2573T>G(p.L858R) | exon 21 | yes | 12 |
|  | c.2609A>G(p.H870R) | exon 21 |  |  |
| P164 | c.2492G>A(p.R831H) | exon 21 | yes | 27 |
|  | c.2573T>G(p.L858R) | exon 21 |  |  |
| P165 | c.2504A>T(p.H835L) | exon 21 | yes | 2 |
|  | c.2497T>G(p.L833V) | exon 21 |  |  |
| P166 | c.2126A>C(p.E709A) | exon 18 | yes | 10 |
|  | c.2156G>C(p.G719A) | exon 18 |  |  |
| P167 | c.2573T>G(p.L858R) | exon 21 | yes | 4 |
|  | c.2560A>G(p.T854A) | exon 21 |  |  |
| P168 | c.2573T>G(p.L858R) | exon 21 | yes | 12 |
|  | c.2609A>G(p.H870R) | exon 21 |  |  |
| P169 | c.2618G>A(p.G873E) | exon 21 | yes | 15 |
|  | c.2573T>G(p.L858R) | exon 21 |  |  |
| P170 | c.2320G>A(p.V774M) | exon 20 | yes | 1 |
|  | c.2318A>T(p.H773L) | exon 20 |  |  |
| P171 | c.2573T>G(p.L858R) | exon 21 | yes | 2 |
|  | c.2579A>T(p.K860I) | exon 21 |  |  |
| P172 | c.2117T>C(p.I706T) | exon 18 | yes | 13 |
|  | c.2156G>C(p.G719A) | exon 18 |  |  |
| P173 | c.2573T>G(p.L858R) | exon 21 | yes | 27 |
|  | c.2492G>A(p.R831H) | exon 21 |  |  |
| P174 | c.2573T>G(p.L858R) | exon 21 | yes | 13 |
|  | c.2612C>A(p.A871E) | exon 21 |  |  |
| P175 | c.2512C>G(p.L838V) | exon 21 | yes | 20 |
|  | c.2573T>G(p.L858R) | exon 21 |  |  |
| P176 | c.2573T>G(p.L858R) | exon 21 | yes | 24 |
|  | c.2500G>T(p.V834L) | exon 21 |  |  |
| P177 | c.2305G>T(p.V769L) | exon 20 | yes | 1 |
|  | c.2303G>T(p.S768I) | exon 20 |  |  |
| P178 | c.2573T>G(p.L858R) | exon 21 | yes | 14 |
|  | c.2530C>G(p.L844V) | exon 21 |  |  |
| P179 | c.2125G>A(p.E709K) | exon 18 | yes | 10 |
|  | c.2156G>C(p.G719A) | exon 18 |  |  |
| P180 | c.2156G>C(p.G719A) | exon 18 | yes | 12 |
|  | c.2120T>G(p.L707W) | exon 18 |  |  |
| P181 | c.2499G>C(p.L833F) | exon 21 | yes | 25 |
|  | c.2573T>G(p.L858R) | exon 21 |  |  |
| P182 | c.2320G>A(p.V774M) | exon 20 | yes | 4 |
|  | c.2332C>G(p.L778V) | exon 20 |  |  |
| P183 | c.2303G>T(p.S768I) | exon 20 | yes | 6 |
|  | c.2320G>A(p.V774M) | exon 20 |  |  |
| P184 | c.2318A>T(p.H773L) | exon 20 | yes | 1 |
|  | c.2320G>A(p.V774M) | exon 20 |  |  |
| P185 | c.2500G>T(p.V834L) | exon 21 | yes | 24 |
|  | c.2573T>G(p.L858R) | exon 21 |  |  |
| P186 | c.2500G>T(p.V834L) | exon 21 | yes | 24 |
|  | c.2573T>G(p.L858R) | exon 21 |  |  |
| P187 | c.2590G>C(p.A864P) | exon 21 | yes | 6 |
|  | c.2573T>G(p.L858R) | exon 21 |  |  |
| P188 | c.2140A>G(p.K714E) | exon 18 | yes | 5 |
|  | c.2155G>T(p.G719C) | exon 18 |  |  |
| P189 | c.2573T>G(p.L858R) | exon 21 | yes | 24 |
|  | c.2500G>T(p.V834L) | exon 21 |  |  |
| P190 | c.2573T>G(p.L858R) | exon 21 | yes | 13 |
|  | c.2612C>G(p.A871G) | exon 21 |  |  |
| P191 | c.2573T>G(p.L858R) | exon 21 | yes | 24 |
|  | c.2500G>T(p.V834L) | exon 21 |  |  |
| P192 | c.2504A>T(p.H835L) | exon 21 | yes | 2 |
|  | c.2497T>G(p.L833V) | exon 21 |  |  |
| P193 | c.2573_2574delTGinsGT(p.L858R) | exon 21 | yes | 2 |
|  | c.2579A>T(p.K860I) | exon 21 |  |  |
| P194 | c.2573T>G(p.L858R) | exon 21 | no | 21 |
|  | c.2509G>A(p.D837N) | exon 21 |  |  |
| P195 | c.2573T>G(p.L858R) | exon 21 | yes | 24 |
|  | c.2500G>T(p.V834L) | exon 21 |  |  |
| P196 | c.2156G>C(p.G719A) | exon 18 | yes | 10 |
|  | c.2126A>C(p.E709A) | exon 18 |  |  |
| P197 | c.2497T>G(p.L833V) | exon 21 | yes | 25 |
|  | c.2573T>G(p.L858R) | exon 21 |  |  |
| P198 | c.2126A>C(p.E709A) | exon 18 | yes | 10 |
|  | c.2155G>A(p.G719S) | exon 18 |  |  |
| P199 | c.2303G>T(p.S768I) | exon 20 | yes | 1 |
|  | c.2305G>T(p.V769L) | exon 20 |  |  |
| P200 | c.2500G>T(p.V834L) | exon 21 | yes | 24 |
|  | c.2573T>G(p.L858R) | exon 21 |  |  |
| P201 | c.2582T>A(p.L861Q) | exon 21 | yes | 28 |
|  | c.2498T>G(p.L833W) | exon 21 |  |  |
| P202 | c.2320G>A(p.V774M) | exon 20 | yes | 1 |
|  | c.2318A>T(p.H773L) | exon 20 |  |  |
| P203 | c.2126A>C(p.E709A) | exon 18 | yes | 10 |
|  | c.2156G>C(p.G719A) | exon 18 |  |  |
| P204 | c.2612C>A(p.A871E) | exon 21 | yes | 13 |
|  | c.2573T>G(p.L858R) | exon 21 |  |  |
| P205 | c.2609A>G(p.H870R) | exon 21 | yes | 12 |
|  | c.2573T>G(p.L858R) | exon 21 |  |  |
| P206 | c.2500G>T(p.V834L) | exon 21 | yes | 24 |
|  | c.2573T>G(p.L858R) | exon 21 |  |  |
| P207 | c.2573T>G(p.L858R) | exon 21 | yes | 15 |
|  | c.2618G>A(p.G873E) | exon 21 |  |  |
| P208 | c.2590G>C(p.A864P) | exon 21 | yes | 6 |
|  | c.2573T>G(p.L858R) | exon 21 |  |  |
| P209 | c.2573T>G(p.L858R) | exon 21 | yes | 24 |
|  | c.2500G>C(p.V834L) | exon 21 |  |  |
| P210 | c.2573T>G(p.L858R) | exon 21 | yes | 24 |
|  | c.2500G>T(p.V834L) | exon 21 |  |  |
| P211 | c.2497T>G(p.L833V) | exon 21 | yes | 25 |
|  | c.2573T>G(p.L858R) | exon 21 |  |  |
| P212 | c.2492G>A(p.R831H) | exon 21 | yes | 27 |
|  | c.2573T>G(p.L858R) | exon 21 |  |  |
| P213 | c.2154_2155delGGinsTT(p.G719C) | exon 18 | yes | 10 |
|  | c.2126A>T(p.E709V) | exon 18 |  |  |
| P214 | c.2527G>A(p.V843I) | exon 21 | no | 15 |
|  | c.2573T>G(p.L858R) | exon 21 |  |  |
| P215 | c.2335_2336delGGinsTT(p.G779F) | exon 20 | yes | 3 |
|  | c.2326C>A(p.R776S) | exon 20 |  |  |
| P216 | c.2582T>G(p.L861R) | exon 21 | yes | 28 |
|  | c.2499G>T(p.L833F) | exon 21 |  |  |
| P217 | c.2305G>T(p.V769L) | exon 20 | yes | 1 |
|  | c.2303G>T(p.S768I) | exon 20 |  |  |
| P218 | c.2512C>G(p.L838V) | exon 21 | yes | 23 |
|  | c.2582T>A(p.L861Q) | exon 21 |  |  |
| P219 | c.2497T>G(p.L833V) | exon 21 | yes | 25 |
|  | c.2573T>G(p.L858R) | exon 21 |  |  |
| P220 | c.2500G>T(p.V834L) | exon 21 | yes | 24 |
|  | c.2573T>G(p.L858R) | exon 21 |  |  |
| P221 | c.2573T>G(p.L858R) | exon 21 | yes | 25 |
|  | c.2499G>T(p.L833F) | exon 21 |  |  |
| P222 | c.2573T>G(p.L858R) | exon 21 | yes | 13 |
|  | c.2533G>C(p.V845L) | exon 21 |  |  |
| P223 | c.2618G>A(p.G873E) | exon 21 | yes | 15 |
|  | c.2573T>G(p.L858R) | exon 21 |  |  |
| P224 | c.2573T>G(p.L858R) | exon 21 | yes | 25 |
|  | c.2497T>G(p.L833V) | exon 21 |  |  |
| P225 | c.2497T>G(p.L833V) | exon 21 | yes | 25 |
|  | c.2573T>G(p.L858R) | exon 21 |  |  |
| P226 | c.2594A>G(p.E865G) | exon 21 | yes | 7 |
|  | c.2573T>G(p.L858R) | exon 21 |  |  |
| P227 | c.2497T>G(p.L833V) | exon 21 | yes | 2 |
|  | c.2504A>T(p.H835L) | exon 21 |  |  |
| P228 | c.2588G>A(p.G863D) | exon 21 | yes | 5 |
|  | c.2573T>G(p.L858R) | exon 21 |  |  |
| P229 | c.2497T>G(p.L833V) | exon 21 | yes | 2 |
|  | c.2504A>T(p.H835L) | exon 21 |  |  |
| P230 | c.2612C>G(p.A871G) | exon 21 | yes | 13 |
|  | c.2573T>G(p.L858R) | exon 21 |  |  |
| P231 | c.2303G>T(p.S768I) | exon 20 | yes | 1 |
|  | c.2305G>T(p.V769L) | exon 20 |  |  |
| P232 | c.2126A>G(p.E709G) | exon 18 | yes | 10 |
|  | c.2155G>A(p.G719S) | exon 18 |  |  |
| P233 | c.2504A>T(p.H835L) | exon 21 | yes | 2 |
|  | c.2497T>G(p.L833V) | exon 21 |  |  |
| P234 | c.2303G>T(p.S768I) | exon 20 | yes | 6 |
|  | c.2320G>A(p.V774M) | exon 20 |  |  |
| P235 | c.2155G>A(p.G719S) | exon 18 | yes | 10 |
|  | c.2126A>C(p.E709A) | exon 18 |  |  |
| P236 | c.2303G>T(p.S768I) | exon 20 | yes | 6 |
|  | c.2320G>A(p.V774M) | exon 20 |  |  |
| P237 | c.2156G>C(p.G719A) | exon 18 | yes | 10 |
|  | c.2126A>C(p.E709A) | exon 18 |  |  |
| P238 | c.2573T>G(p.L858R) | exon 21 | yes | 24 |
|  | c.2500G>T(p.V834L) | exon 21 |  |  |
| P239 | c.2560A>G(p.T854A) | exon 21 | yes | 4 |
|  | c.2573T>G(p.L858R) | exon 21 |  |  |
| P240 | c.2500G>T(p.V834L) | exon 21 | yes | 24 |
|  | c.2573T>G(p.L858R) | exon 21 |  |  |
| P241 | c.2612_2613delCAinsGC(p.A871G) | exon 21 | yes | 13 |
|  | c.2573T>G(p.L858R) | exon 21 |  |  |
| P242 | c.2497T>G(p.L833V) | exon 21 | yes | 25 |
|  | c.2573T>G(p.L858R) | exon 21 |  |  |
| P243 | c.2155G>A(p.G719S) | exon 18 | yes | 10 |
|  | c.2126A>C(p.E709A) | exon 18 |  |  |
| P244 | c.2572C>A(p.L858M) | exon 21 | yes | 3 |
|  | c.2582T>G(p.L861R) | exon 21 |  |  |
| P245 | c.2318A>T(p.H773L) | exon 20 | yes | 1 |
|  | c.2320G>A(p.V774M) | exon 20 |  |  |
| P246 | c.2499G>T(p.L833F) | exon 21 | yes | 28 |
|  | c.2582T>G(p.L861R) | exon 21 |  |  |
| P247 | c.2504A>T(p.H835L) | exon 21 | yes | 2 |
|  | c.2497T>G(p.L833V) | exon 21 |  |  |
| P248 | c.2582T>A(p.L861Q) | exon 21 | yes | 3 |
|  | c.2572C>A(p.L858M) | exon 21 |  |  |
| P249 | c.2573T>G(p.L858R) | exon 21 | yes | 20 |
|  | c.2512C>G(p.L838V) | exon 21 |  |  |
| P250 | c.2320G>A(p.V774M) | exon 20 | yes | 1 |
|  | c.2318A>T(p.H773L) | exon 20 |  |  |
| P251 | c.2573T>G(p.L858R) | exon 21 | yes | 13 |
|  | c.2612C>A(p.A871E) | exon 21 |  |  |
| P252 | c.2497T>G(p.L833V) | exon 21 | yes | 25 |
|  | c.2573T>G(p.L858R) | exon 21 |  |  |
| P253 | c.2500G>T(p.V834L) | exon 21 | yes | 24 |
|  | c.2573T>G(p.L858R) | exon 21 |  |  |
| P254 | c.2579A>T(p.K860I) | exon 21 | yes | 2 |
|  | c.2573T>G(p.L858R) | exon 21 |  |  |
| P255 | c.2496_2497inv(p.L833V) | exon 21 | yes | 2 |
|  | c.2504A>T(p.H835L) | exon 21 |  |  |
| P256 | c.2497T>G(p.L833V) | exon 21 | yes | 2 |
|  | c.2504A>T(p.H835L) | exon 21 |  |  |
| P257 | c.2156G>C(p.G719A) | exon 18 | yes | 10 |
|  | c.2125G>A(p.E709K) | exon 18 |  |  |
| P258 | c.2126A>C(p.E709A) | exon 18 | yes | 10 |
|  | c.2155G>T(p.G719C) | exon 18 |  |  |
| P259 | c.2497T>G(p.L833V) | exon 21 | yes | 25 |
|  | c.2573T>G(p.L858R) | exon 21 |  |  |
| P260 | c.2527G>A(p.V843I) | exon 21 | yes | 15 |
|  | c.2573T>G(p.L858R) | exon 21 |  |  |
| P261 | c.2573T>G(p.L858R) | exon 21 | yes | 25 |
|  | c.2497T>G(p.L833V) | exon 21 |  |  |
| P262 | c.2305G>T(p.V769L) | exon 20 | yes | 1 |
|  | c.2303G>T(p.S768I) | exon 20 |  |  |
| P263 | c.2504A>T(p.H835L) | exon 21 | yes | 2 |
|  | c.2497T>G(p.L833V) | exon 21 |  |  |
| P264 | c.2573T>G(p.L858R) | exon 21 | yes | 24 |
|  | c.2500G>C(p.V834L) | exon 21 |  |  |
| P265 | c.2582T>A(p.L861Q) | exon 21 | yes | 28 |
|  | c.2498T>G(p.L833W) | exon 21 |  |  |
| P266 | p.G719S (c.G2155A) | exon 18 | yes | 10 |
|  | p.E709A (c.A2126C) | exon 18 |  |  |
| P267 | p.V834L (c.G2500T) | exon 21 | yes | 1 |
|  | p.L833F (c.G2499T) | exon 21 |  |  |
| P268 | p.S768I (c.G2303T) | exon 20 | yes | / |
|  | p.V769L (c.G2305T) | exon 20 |  |  |
|  | p.A763G (c.C2288G) | exon 20 |  |  |
| P269 | c.2500G>T(p.V834L) | exon 21 | yes | / |
|  | c.2575G>T(p.A859S) | exon 21 |  |  |
|  | c.2573_2574delTGinsGC(p.L858R) | exon 21 |  |  |
| P270 | c.2126A>C(p.E709A) | exon 18 | yes | 10 |
|  | c.2155G>A(p.G719S) | exon 18 |  |  |
| P271 | c.2573T>G(p.L858R) | exon 21 | yes | 10 |
|  | c.2543C>T(p.P848L) | exon 21 |  |  |
| P272 | c.2155G>T(p.G719C) | exon 18 | yes | 10 |
|  | c.2126A>C(p.E709A) | exon 18 |  |  |
| P273 | c.2497T>G(p.L833V) | exon 21 | yes | 2 |
|  | c.2504A>T(p.H835L) | exon 21 |  |  |
| P274 | c.2148A>C(p.K716N) | exon 18 | yes | / |
|  | c.2156G>C(p.G719A) | exon 18 |  |  |
|  | c.2141A>G(p.K714R) | exon 18 |  |  |
|  | c.2137A>C(p.K713Q) | exon 18 |  |  |
| P275 | c.2573T>G(p.L858R) | exon 21 | yes | 25 |
|  | c.2497T>G(p.L833V) | exon 21 |  |  |
| P276 | c.2582T>A(p.L861Q) | exon 21 | yes | 3 |
|  | c.2573T>A(p.L858Q) | exon 21 |  |  |
| P277 | c.2117T>C(p.I706T) | exon 18 | yes | / |
|  | c.2159C>T(p.S720F) | exon 18 |  |  |
|  | c.2156G>C(p.G719A) | exon 18 |  |  |
| P278 | c.3027C>A(p.D1009E) | exon 25 | yes | / |
|  | c.3029T>G(p.V1010G) | exon 25 |  |  |
|  | c.3032T>G(p.V1011G) | exon 25 |  |  |
| P279 | c.2497T>G(p.L833V) | exon 21 | yes | 25 |
|  | c.2573T>G(p.L858R) | exon 21 |  |  |
| P280 | c.2573T>G(p.L858R) | exon 21 | no | 3 |
|  | c.2582T>A(p.L861Q) | exon 21 |  |  |
| P281 | c.2148A>C(p.K716N) | exon 18 | yes | 3 |
|  | c.2156G>C(p.G719A) | exon 18 |  |  |
| P282 | c.2146A>C(p.K716Q) | exon 18 | yes | / |
|  | c.2154_2155delinsTT(p.G719C) | exon 18 |  |  |
|  | c.2126A>T(p.E709V) | exon 18 |  |  |

**Fig. S1** The type of compound *EGFR* mutations and the concurrent genetic alterations. (**A**) Different combinations of *EGFR* mutations in patients with double *EGFR* mutations (n=998). Patients with more than 2 *EGFR* mutations were not included in the analysis. The red color in the oncoprint plot indicates the presence of the corresponding combination of *EGFR* mutations. (**B**) The genetic profile of patients with different types of compound *EGFR* mutations. Patients’ samples that were characterized by targeted NGS of 139 key lung cancer-related genes were included in the analysis (n=720).


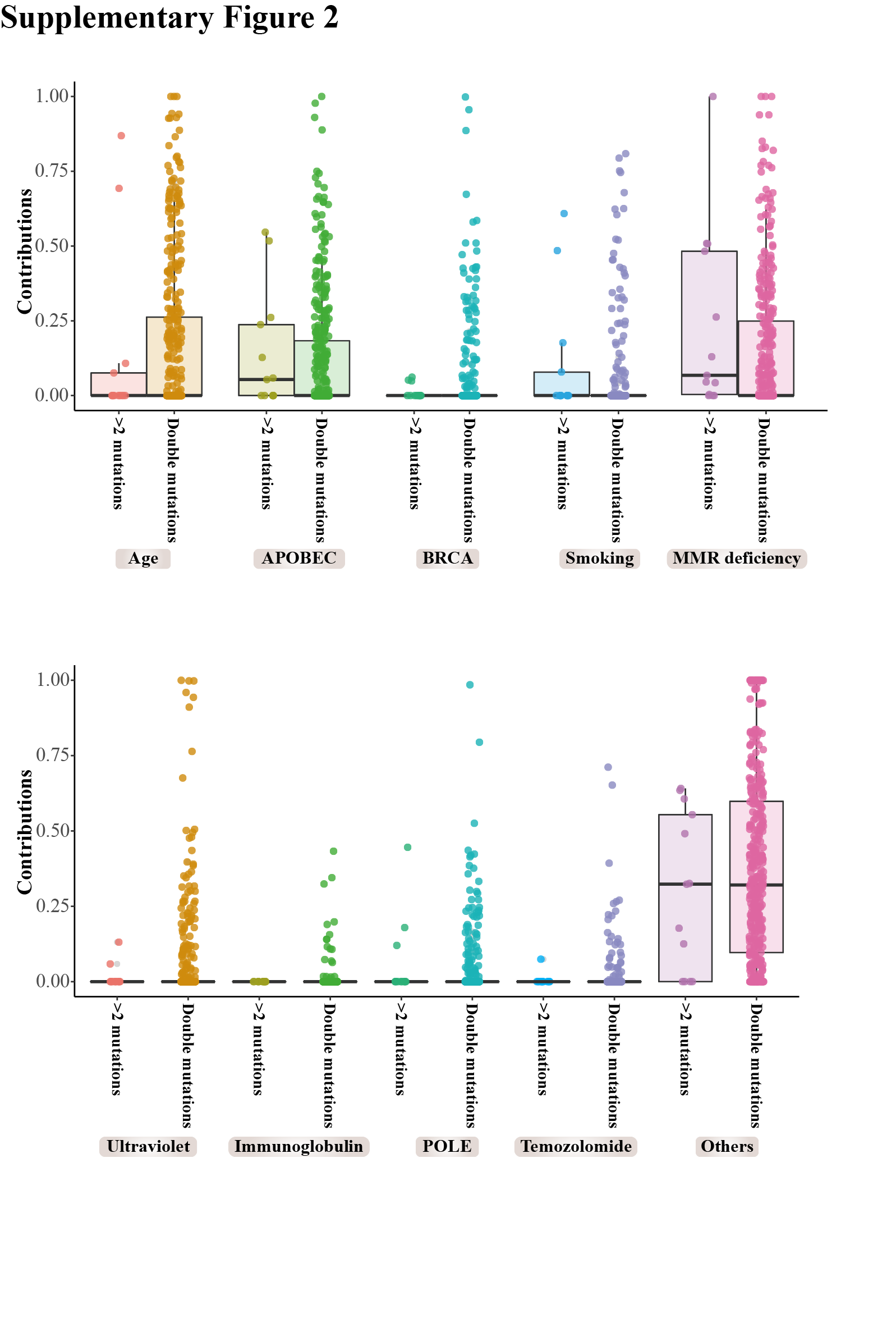


**Fig. S2** The mutational signature analysis for patients with different numbers of *EGFR* mutations. Patients whose baseline tumor tissue samples were characterized by large panel targeted sequencing of 425 cancer-relevant genes were included in the analysis (n=408).


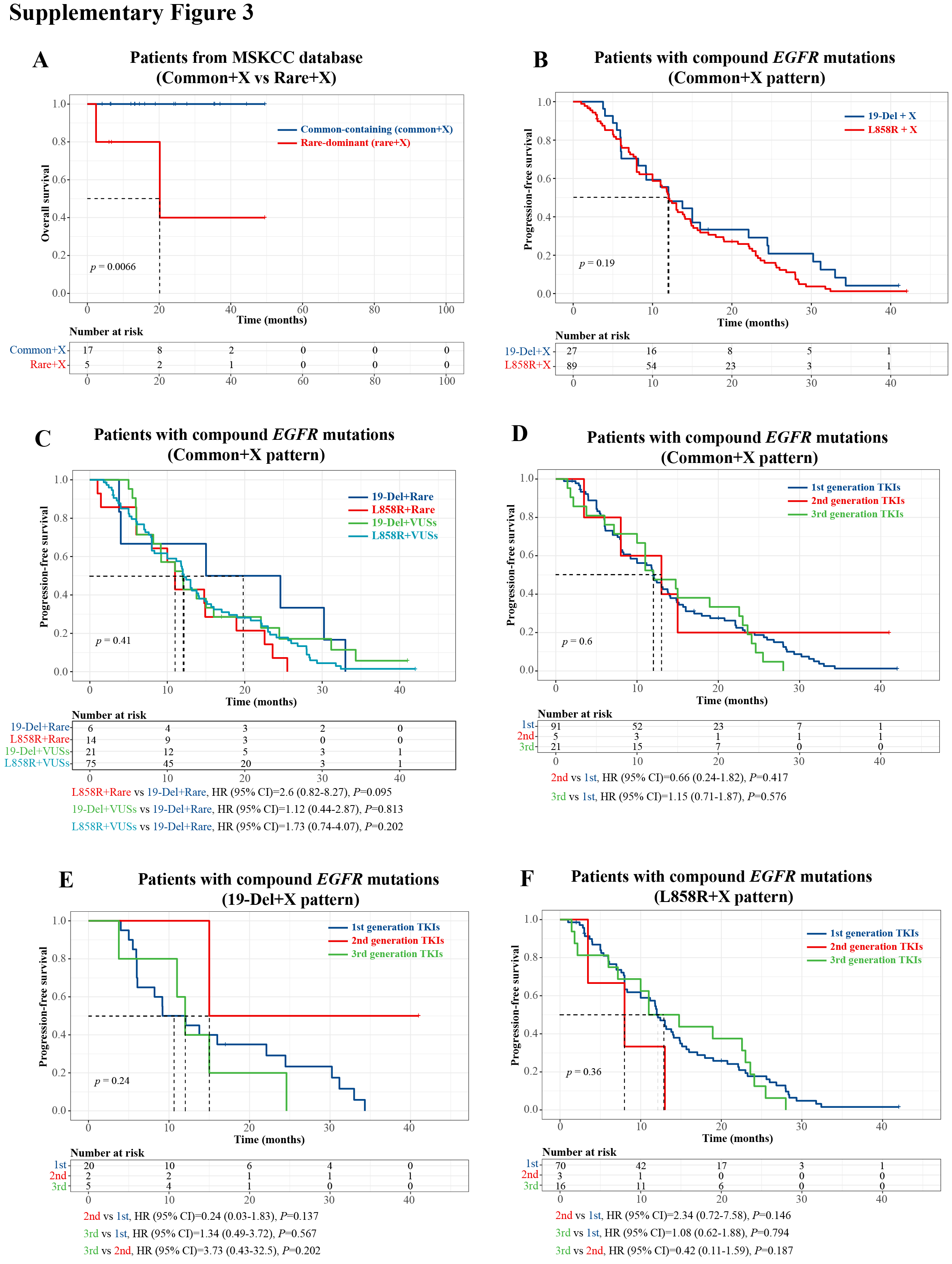


**Fig. S3** The correlation between the common *EGFR* mutation-containing subtype and patients’ prognosis to first-line EGFR TKIs. (**A**) Kaplan-Meier curve of overall survival in patients obtained from the Memorial Sloan Kettering Cancer Center (MSKCC) database (n=22) in strata of the type of compound *EGFR* mutation. (**B**) Kaplan-Meier curve of progression-free survival in patients with common *EGFR* mutation-containing subtypes in strata of the type of common *EGFR* mutation. One patient with the concurrent *EGFR* L858R and 19-Del mutations was not included in the analysis. (**C**) Kaplan-Meier curve of progression-free survival in patients with common *EGFR* mutation-containing subtypes in strata of various combinations of compound *EGFR* mutations. One patient with the concurrent *EGFR* L858R and 19-Del mutations was not included in the analysis. (**D**) Kaplan-Meier curve of progression-free survival in patients with common *EGFR* mutation-containing subtypes in strata of different generations of first-line EGFR TKIs. (**E**) Kaplan-Meier curve of progression-free survival in patients with *EGFR* 19-Del-containing subtype in strata of different generations of first-line EGFR TKIs. (**F**) Kaplan-Meier curve of progression-free survival in patients with *EGFR* L858R-containing subtype in strata of different generations of first-line EGFR TKIs. Log‐rank test with *P*-value<0.05 was considered to be statistically significant (**P*<0.05, ***P*<0.01, ****P*<0.001).


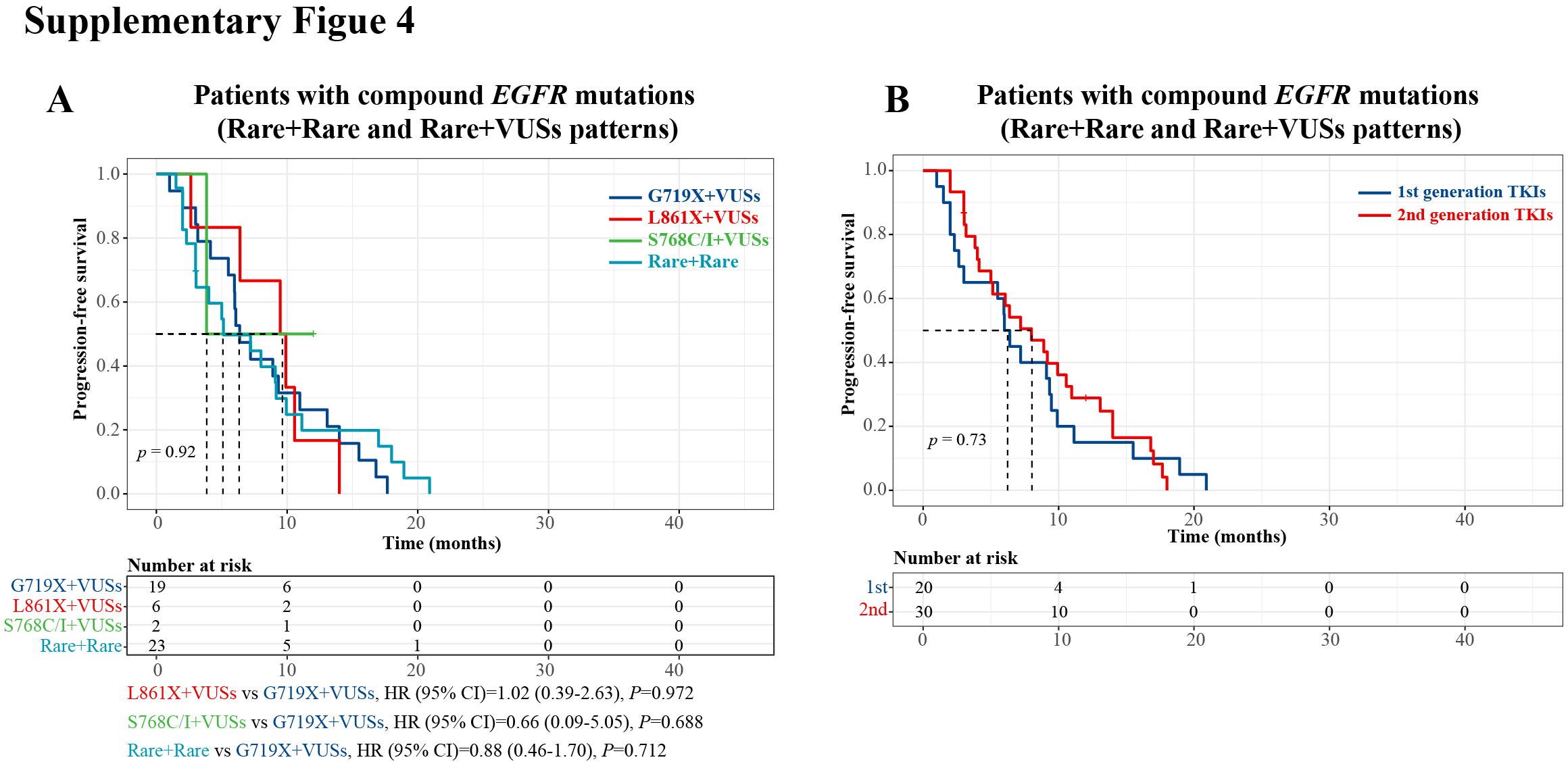


**Fig. S4** The correlation between the rare *EGFR* mutation-dominant subtype and patients’ prognosis to first-line EGFR TKIs. (**A**) Kaplan-Meier curve of progression-free survival in patients with rare *EGFR* mutation-dominant subtypes in strata of different combinations of compound *EGFR* mutations. Patients with more than 2 EGFR mutations were not included in the analysis (n=1). (**B**) Kaplan-Meier curve of progression-free survival in patients with rare *EGFR* mutation-dominant subtype in strata of different generations of first-line EGFR TKIs. Patients with more than 2 *EGFR* mutations were not included in the analysis (n=1).


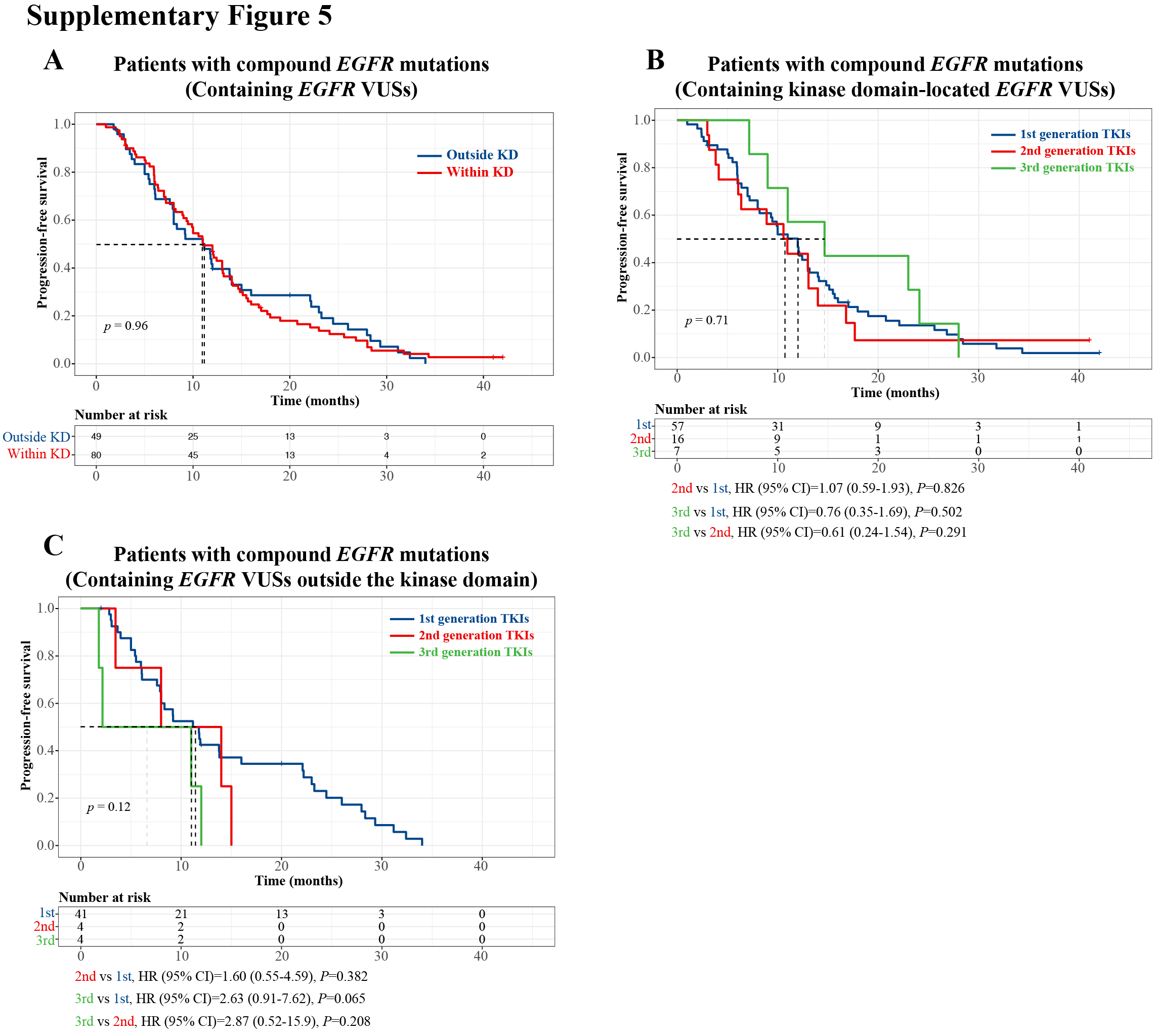


**Fig. S5** The correlation between the *EGFR* VUSs-containing subtype and patients’ prognosis to first-line EGFR TKIs. (**A**) Kaplan-Meier curve of progression-free survival in patients with *EGFR* VUSs-containing subtypes in strata of different locations of *EGFR* VUSs. (**B**) Kaplan-Meier curve of progression-free survival in patients with *EGFR* VUSs (KD+)-containing subtype in strata of different generations of first-line EGFR TKIs. (**C**) Kaplan-Meier curve of progression-free survival in patients with *EGFR* VUSs (KD-)-containing subtype in strata of different generations of first-line EGFR TKIs. KD+, within the EGFR kinase domain; KD-, outside the EGFR kinase domain.


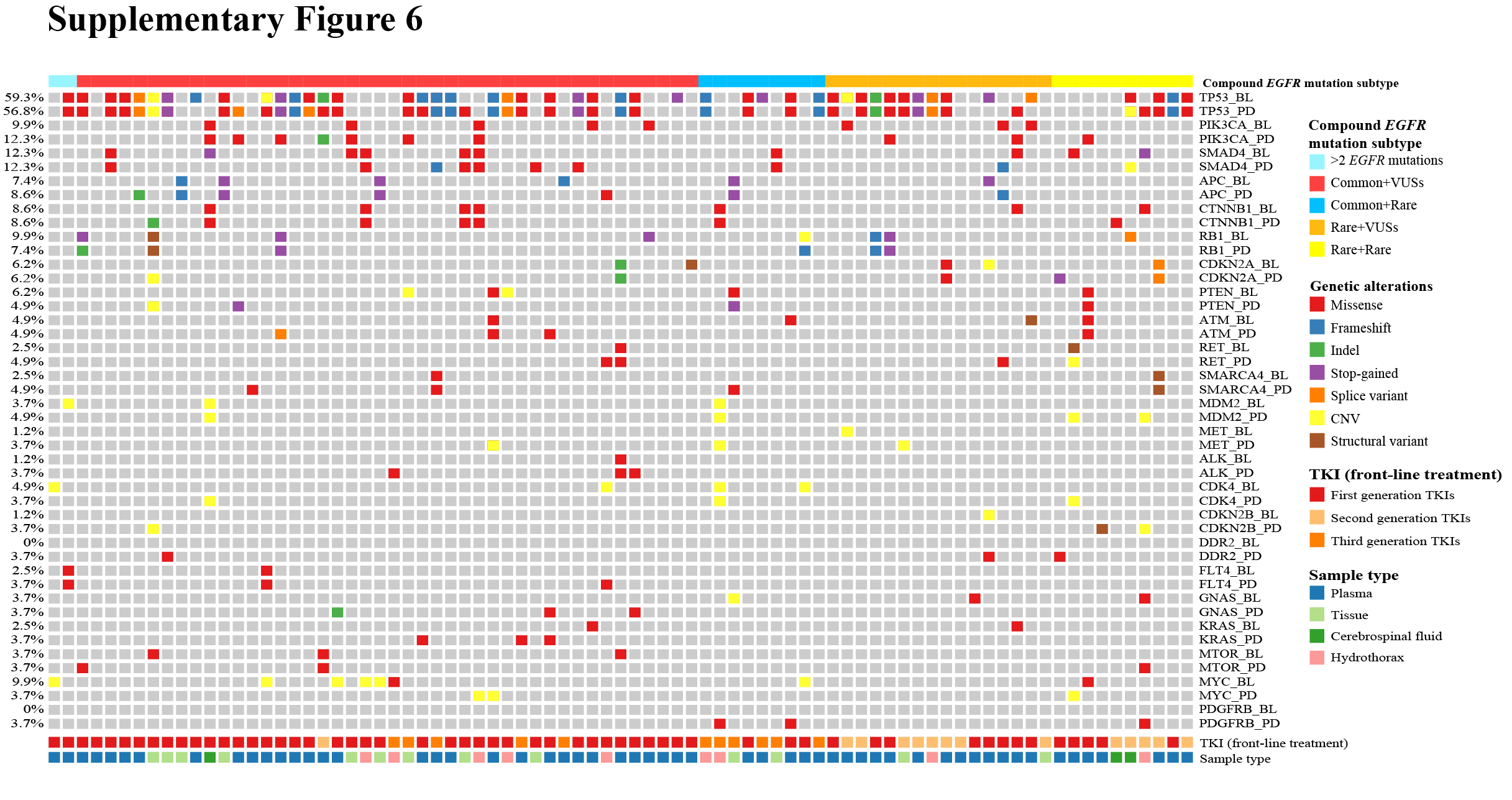


**Fig. S6** The difference of the genetic profile between the baseline sample and the paired PD samples (n=95), stratified by different compound *EGFR* mutation subtypes. BL, baseline; PD, progressive disease.
